# Optomechanical motions of gold dimer’s spin, rotation and revolution manipulated by bessel beam

**DOI:** 10.1038/s41598-024-77413-7

**Published:** 2024-11-04

**Authors:** Chao-Kang Liu, Yun-Cheng Ku, Mao-Kuen Kuo, Jiunn-Woei Liaw

**Affiliations:** 1 Institute of Applied Mechanics, National Taiwan University, Taipei, Taiwan; 2 Department of Mechanical Engineering, Chang Gung University, Taipei, Taiwan; 3 Department of Mechanical Engineering, Ming Chi University of Technology, Taipei, Taiwan; 4 Proton and Radiation Therapy Center, Gung Memorial Hospital, Linkou Chang, Taiwan

**Keywords:** Bessel beam, Orbital angular momentum, Spin angular momentum, Optical binding, MMP, Orbital revolution, Neuroscience, Nanoscience and technology, Optics and photonics

## Abstract

The optomechanical motion of a gold nanoparticle (GNP) dimer—a pair of optically bound GNPs—in fluid, manipulated by a Bessel beam, is theoretically studied using the multiple multipole (MMP) method. Since a Bessel beam possesses orbital angular momentum (OAM) and spin angular momentum (SAM) simultaneously, complicated rigid-body motions of the dimer can be induced. The mechanism involves the equilibrium between the optical force with the reactive drag force exerted by the fluid. Our results demonstrate that the dimer rotates around its center of mass (COM), while the COM performs an orbital revolution around the optical axis. Additionally, each individual GNP undergoes spinning. The directions of the GNPs’ spin and the orbital revolution of COM depend on the handedness and the order (topological charge) of Bessel beam, respectively. Nevertheless, the rotation direction of the dimer depends on the size of GNP. In the case of a smaller dimer, the direction of dimer’s rotation with respect to the COM is consistent with the handedness of the light. Conversely, a larger dimer performs a reverse rotation, accompanied by a precession during the orbital revolution. There are multiple turning points in the radius of the GNP for the alternating rotation of the dimer caused by positive or negative optical torque. Our finding may provide an insight to the optomechanical manipulation of optical vortexes on the motions of GNP clusters.

## Introduction

In recent decades, the motions of microparticles or nanoparticles manipulated by light through the light-matter interaction has gained significant attention^1–9^. In particular, the study of using plasmon-enhanced optical force to manipulate gold nanoparticles (GNPs) or silver nanoparticles (SNPs) for optical tweezers attracted a lot of attention^1,5–16^. For example, a laser beam, a linearly polarized light (LPL), can induce an optical binding for a pair of GNPs with a stable distance of integer multiples of the effective wavelength of light in the medium, where the central line of the dimer is perpendicular to the polarization of LPL^11,12^. In addition, a circularly polarized light (CPL) can induce the spin of a single GNP or gold nanorod (GNR) significantly due to the absorption of photon’s spin angular momentum (SAM)^1,3,13–15^. Although a CPL has only SAM but without orbital angular momentum (OAM) intrinsically, CPL may induce the optical binding between two individual GNPs to form a dimer with a stable distance, and drives them to rotate around their center of mass (COM) accompanied with individual spinning^16^. Of interesting, the rotation’s direction of dimer not only depends on the handedness of CPL but also the size of GNPs^17–19^. For a smaller GNP/SNP dimer, the rotational direction is always consistent with the handedness of CPL. In contrast, for a larger GNP/SNP dimer a reverse rigid-body rotation around the COM may be induced due to the negative optical torque caused by higher-order modes^17–19^. Moreover, using CPL to manipulate multiple SNPs, GNPs or GNRs have also been studied^4,7,15,18,20^. For example, the planetary gear motion of optical bound hexagonal lattice composed of multiply SNPs driven by CPL were investigated by Refs^7,15^.

Recently, the study of using an optical vortex, e.g. Laguerre-Gaussian beam or Bessel beam, to manipulate dielectric microparticles, GNPs or SNPs has also drawn considerable attention^21–39^. Since an optical vortex, a structured light, possesses both SAM and OAM simultaneously, the optomechanical motions of these particles become more versatile. For example, utilizing Bessel beam to capture a single GNP or GNR onto an orbit to perform an orbital revolution with spinning^25,26^. The observation of spin–orbit interaction (SOI) of the electromagnetic (EM) field through the light interacting with these plasmonic nanostructures is noted; a part of SAM of optical vortex may be converted into OAM via the light-matter interaction^23,26^. Moreover, a plasmonic dimer, two optically bound SNPs, may perform an orbital motion in an optical field with transverse phase gradient^5,28^.

**Fig. 1:**
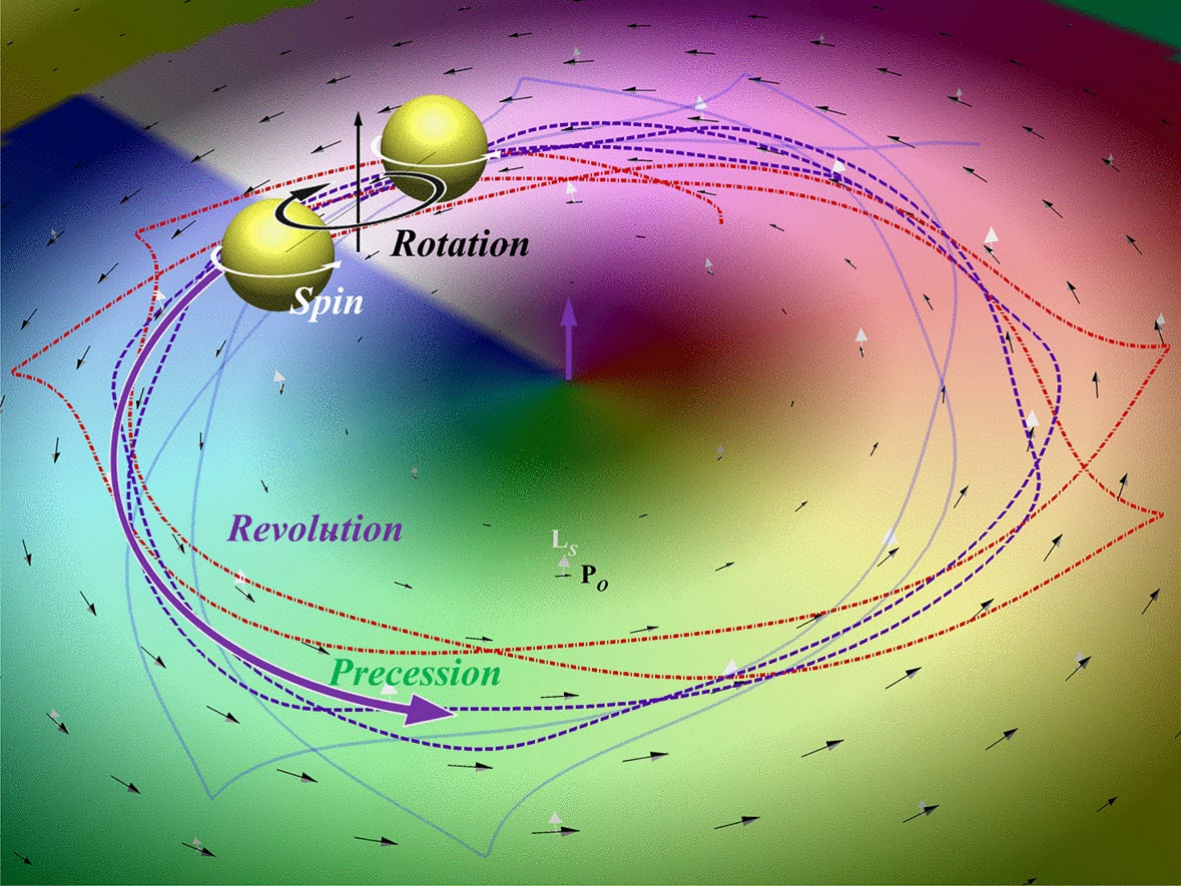
Configuration of optically bound GNP dimer’s motions of spin, rotation and orbital revolution induced by a Bessel beam. If the directions of rotation and revolution are opposite, a precession is induced in COM’s orbital revolution.

In this paper, we focus on the study of the motions of two identical GNPs manipulated by different-order Bessel beams. We use the multiple multipole (MMP) method to simulate the coupled EM field of two discrete GNPs interacting with an incident Bessel beam, and then utilize the results to obtain the optical force and torque upon individual GNP^14,16,23,26^. Subsequently, we calculate the trajectories of these GNPs based on their equations of motion in terms of the optical force and the drag fore of fluid. Furthermore, the trajectory of the two GNPs’ COM will be analyzed. In addition, the terminal spin of each GNP due to the balance of the optical torque with the drag torque will be investigated. Figure 1 illustrates the configuration of the motions (spin, rotation and orbital revolution) of a GNP dimer manipulated by a Bessel beam. Herein, we assume that the sizes of both spherical GNPs are identical and the medium is water with a refractive index of *n* = 1.33. The trajectories of the two GNPs and their COM are depicted in Fig. 1 to illustrate our findings. In this graphical schematic of Fig. 1, we demonstrate that due to optical binding, the two GNPs will ultimately form a dimer, stabilizing at a distance of approximately *λ/n*, irrespective of their initial positions. Additionally, the dimer may undergo rotation around its COM, which in turn experiences an orbital revolution around the *z*-axis, aligning with the optical axis of a normally incident Bessel beam. The size-dependent rigid-body rotation of the dimer will also be studied. Moreover, a precession in COM’s orbital revolution may be induced, if the directions of rotation and revolution are opposite.

## Method

We use the MMP method to simulate the coupled EM field of two discrete GNPs interacting with an incident Bessel beam, and utilize the results to obtain the optical force upon individual GNP^14,16,23,26^. The optical forces **F**_*i*_ (*i* = 1 or 2) exerted on the two discrete GNPs can be obtained by calculating the surface integral of Maxwell’s stress tensor **T** in terms of EM field,

1 $${\bf{F}}_{i}=\int_{S_i}\bf{n}{\cdot}\bf{T}\textit{dS}$$

and

2 $$\bf{T}=\frac{1}{2}\textit{Re}\left({\upepsilon}\bf{E}{\otimes}\overline{\bf{E}}+{\mu}\bf{H}{\otimes}\overline{\bf{H}}-\frac{1}{2}\bf{I}({\upepsilon}\bf{E}{\cdot}\overline{\bf{E}}+{\mu}\bf{H}{\cdot}\overline{\bf{H}})\right)$$

where the upper bar denotes the conjugate and Re is the real part. In Eq. (2), *ε* and *μ* are the permittivity and permeability of the medium, respectively. Subsequently, we calculate the equation of motion of individual GNP in terms of the optical force and the reactive drag fore of fluid. GNPs’ motions obey the equation of motion based on Newton’s second law as follows,

3 $$ m_i\frac{d^2\bf{x}_\textit{i}}{dt^2}=-{\eta}_i\frac{d\bf{x}_\textit{i}}{dt}+\bf{F}_\textit{i}$$

In the above equation, **x**_*i*_ (*i* = 1 or 2) represents the position vector of the *i*-th GNP’s center, and **F**_*i*_ is the optical force upon it, where -*η*_*i*_*d***x**_*i*_/*dt* is the drag force of medium according to Stokes’ first law. Here, the coefficient *η*_*i*_ is 6π*μ*_*v*_*a*_*i*_ with the viscosity *μ*_*v*_ and the radius *a*_*i*_ of the *i*-th GNP. We use the Runge–Kutta method to numerically solve the equations of motion, step by step, considering the optical and drag forces acting on individual GNPs in the medium^23^. In this paper, we neglect the effect of Brownian motion, assuming that the intensity of the Bessel beam is high enough to generate optical forces strong enough to overcome the random forces of Brownian motion. Furthermore, the trajectory of the two GNPs’ COM will be analyzed. The position vector and velocity of COM are defined as.

4 $${\bf{x}}_{c}=\frac{1}{M}\sum_im_i\bf{x}_\textit{i}$$

5 $${\bf{v}}_{c}=\frac{1}{M}\sum_im_i\frac{d\bf{x}_\textit{i}}{dt}$$

where *M* is the total mass of these GNPs; *M* = Σ *m*_*i*_. The *z* component of the angular speed of orbital revolution Ω_*c*_ is defined as.

6 $$\Omega_c=\frac{{\bf{x}_\textit{c}}\times{\bf{v}_\textit{c}}}{{\mid}\bf{x}_\textit{c}{\mid}^2}{\cdot}\bf{e}_\textit{z}$$

where ${\left|{\mathbf{x}}_{c}\right|}^{2}$ is the radius of the orbit. The* z* component of the angular speed of rotation Ω_*p*_ of the *i*-th GNP *w. r. t.* COM is

7 $$\Omega_p=\frac{{(\bf{x}_\textit{i}-\bf{x}_\textit{c})}\times(\bf{v}_\textit{i}-\bf{v}_\textit{c})}{{\mid}\bf{x}_\textit{i}-\bf{x}_\textit{c}{\mid}^2}{\cdot}\bf{e}_\textit{z}$$

The optical spin torque **M**_*S,i*_ on the *i*-th GNP is defined

8 $$\bf{M}_\textit{S,i}={\int}\bf{r}{\times}(\bf{T}{\cdot}\bf{n})\textit{dS}$$

where **r** is the position vector of any point on the surface of* S*_*i*_ with respect to the center of each GNP. According to Stokes’ second law, the* z* component of the terminal spin angular speed of the* i*-th GNP is calculated by using optical spin torque **M**_*S,i*_

9 $$\omega_{S,i}=\frac{\bf{e}_\textit{z}{\cdot}\bf{M}_\textit{S,i}}{8{\pi}{\mu}_va^3}$$

where *a* = *a*_*i*_. In addition, the optical rotation torque **M**_*O,i*_ with respect to COM on the *i*-th GNP is defined

10 $$\bf{M}_\textit{O,i}=(\bf{x}_\textit{i}-\bf{x}_\textit{c})\times{\int}_\textit{Si}\bf{T}{\cdot}\bf{n}\textit{dS}$$

Based on Eq. (10), the terminal rotation angular speed can be determined by assuming equilibrium between the total optical rotation torque and the total resistant torque caused by the drag forces from the medium upon the two GNPs.

The electric field of an incident right-handed (RH) Bessel beam of the* l*-th order in the cylindrical coordinates,

11 $$\bf{E}^\textit{i}{(\textit{r},\theta, \textit{z})}={\textit{E}_\textit{0}}\left(\textit{j}\frac{\textit{k}_\textit{z}}{\textit{k}_\textit{r}}\textit{J}_\textit{l}{(\textit{k}_\textit{r}\textit{r})}\textit{e}^{\textit{jl}\theta}(\bf{e}_\textit{x}+\textit{j}\bf{e}_\textit{y})+\textit{J}_{\textit{l}+1}{(\textit{k}_\textit{r}\textit{r})}\textit{e}^{\textit{j}(\text{l}+1)\theta}\bf{e}_\textit{z}\right)\textit{e}^{\textit{jk}_\textit{2}\textit{z}}$$

where the superscript *i* represents incident light, *E*_0_ is the amplitude, and *J*_*l*_ is the Bessel function of the *l*-th order. Here, the time-harmonic factor of Maxwell’s equations is *exp*(-*jωt*); *ω* is the angular frequency. The cone angle *α* of Bessel beam is $\alpha=tan^{-1}(k_r/k_z)$, where the wavenumber *k* is $k^2=k^2_r+k^2_z$. Similarly, the electric field of an incident left-handed (LH) Bessel beam is

12 $$\bf{E}^\textit{i}{(\textit{r},\theta, \textit{z})}={\textit{E}_\textit{0}}\left(\textit{j}\frac{\textit{k}_\textit{z}}{\textit{k}_\textit{r}}\textit{J}_\textit{l}{(\textit{k}_\textit{r}\textit{r})}\textit{e}^{\textit{jl}\theta}(\bf{e}_\textit{x}-\textit{j}\bf{e}_\textit{y})-\textit{J}_{\textit{l}-1}{(\textit{k}_\textit{r}\textit{r})}\textit{e}^{\textit{j}(\text{l}-1)\theta}\bf{e}_\textit{z}\right)\textit{e}^{\textit{jk}_\textit{2}\textit{z}}$$

Throughout this paper, we assume that the two GNPs are confined to a specific *x–y* plane, resulting in 2D motion, and any movement along the *z*-axis is neglected. Additionally, the Bessel beam model we use is non-focused, meaning the incident electromagnetic field is independent of the *z*-axis. In the corresponding experimental setup, the GNPs are confined between two glass plates. Although optical forces, generated by the linear momentum of photons absorbed by the GNPs, include forces in the *z*-direction, the nanoparticles do not move along the *z*-axis. This is because the reaction forces from the glass plates counteract the forces in the* z*-direction, resulting in zero net force along the *z*-axis for each GNP.

## Results and discussion

**Fig. 2:**
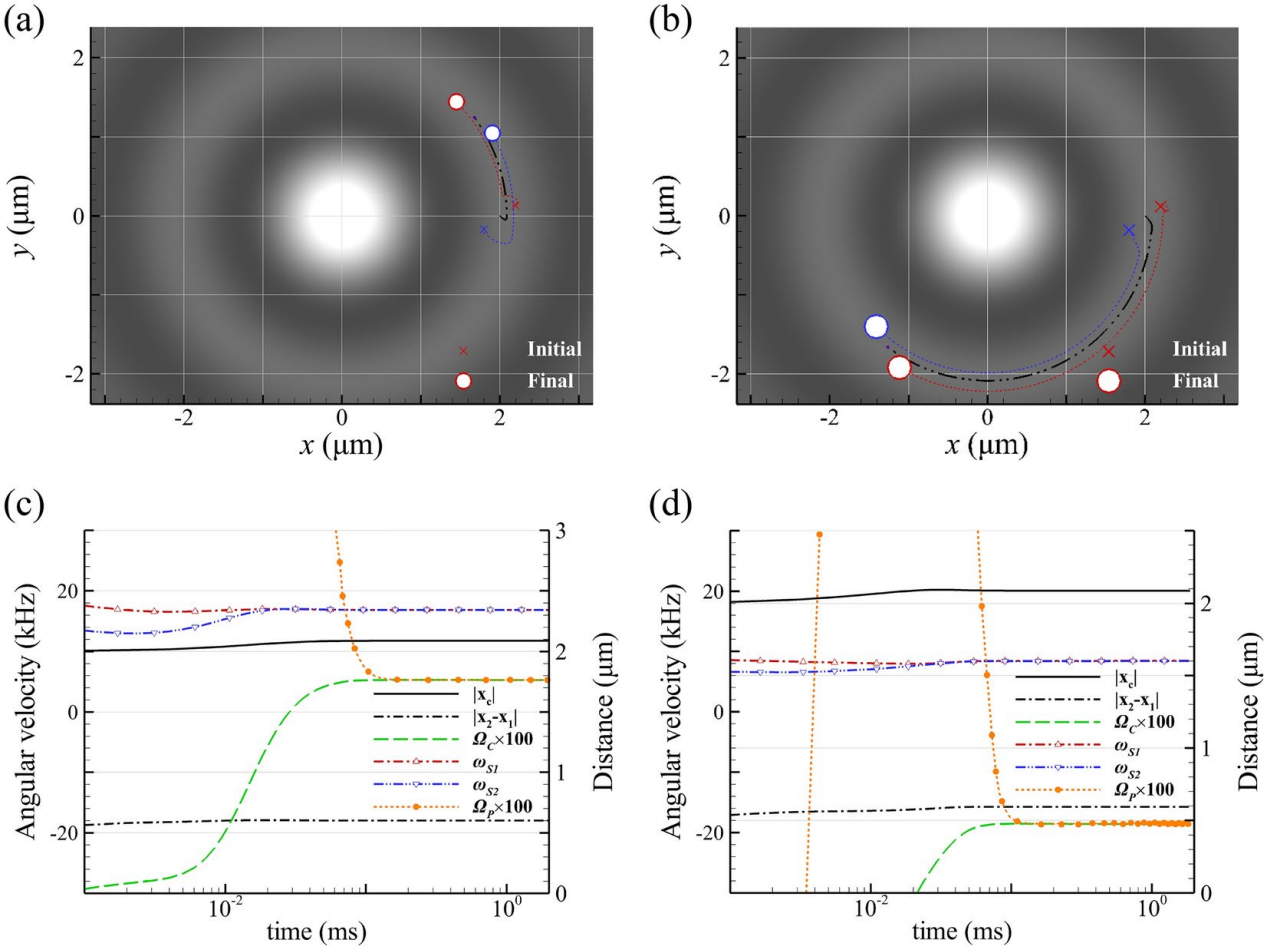
The trajectories of GNP dimer of (**a**) *a* = 100 nm and (**b**) 150 nm, respectively, irradiated by RH Bessel beam of *l* = 0. (**c**) and (**b**) The corresponding orbital radius of COM, and the angular speeds of spin, rotation and revolution of GNPs of *a* = 100 nm and 150 nm, respectively, versus time. The orbital radius of COM is about 2.09 μm for both cases. In (**c**) and (**d**), the black solid line: the revolution radius of COM’s trajectory, and the black dash line: the distance between two GNPs. In (**c**) and (**d**), the value of Ω_*p*_ is too large to exceed the range of the *y*-axis.

The motions of GNP dimers of different sizes manipulated by LH or RH Bessel beams of different orders are studied. The motions of two individual and identical GNPs in water under the irradiation of 800-nm Bessel beams of different orders, *l* = 0, 1 or 2, propagating in *z* direction are simulated by using particles’ dynamic equations of motion in terms of the optical forces, Eq. (3). The optical forces exerted on the two GNPs are determined by calculating the surface integrals of Maxwell’s stress tensors of each GNP, where the EM field is simulated by MMP method^23,26^. The wavelength of light is 800 nm; the effective wavelength in water is about 601 nm. The dielectric constant of gold at a wavelength of 800 nm, cited from Ref^40^., is utilized for the following simulation. The intensity distributions of Bessel beams of *l* = 0, 1 or 2 are plotted in Figure S1 (Supplementary Material), where the radii of the first ring with the peak intensity of these Bessel beams are 2115 nm, 1020 nm and 1690 nm, respectively. Throughout this paper, the amplitude of Bessel beam is $E_0=11.89(\frac{MV}{m})$. Figure 2a shows the trajectory of the two identical GNPs of *a* = 100 nm irradiated by RH Bessel beams of *l* = 0 with a cone angle of *α* = 10°. The result of *a* = 150 nm is shown in Fig. 2b. Our data shows that regardless of the initial positions of the two GNPs they will inevitably become optically bound to form a dimer rotating around their COM. Additionally, the COM simultaneously performs an orbital revolution around the optical axis. The distance between the two GNPs is approximately 600 nm, which is close to the effective wavelength of light in water. The lateral scattering field from two initially separated GNPs provides optical binding to confine them for the formation of a dimer with a fixed distance. In addition, each GNP spins individually. The corresponding orbital radius of COM, angular speeds of spin, rotation and revolution of GNPs are shown in Figs. 2c and 2d. Although the Bessel beam of *l* = 0 only has SAM without OAM, the orbital revolution of dimer’s COM is still induced. It implies that a part of SAM of light is converted into OAM exerted on the two GNPs through the light-matter interaction. For smaller GNPs (*a* = 100 nm), the rotational direction of the dimer is consistent with the handedness of light. The GNP dimer’s rotation is likely caused by the absorption of photon’s SAM. However, the rotational direction of a larger GNP (*a*= 150 nm) dimer is against the direction of SAM, because the negative optical torque is induced. This phenomenon has been discussed in the previous studies^17–19^. The average terminal angular speeds of spin, rotation and revolution of GNP dimer induced by Bessel beams of *l* = 0, 1 and 2 are listed in Table I, when the steady-state motions of the two light-driven GNPs are reached. The dynamic motions of the two GNPs induced by Bessel beam of *l* = 0 are referred to Visualization 1. In contrast, the orbital revolution of a smaller GNP dimer of *a* = 50 nm is nearly absent due to the small scattering cross section, as shown in Figure S2 (Supplementary Material); the SOI effect can almost be neglected. The terminal angular speeds of *a* = 50 nm are listed in Table SI (Supplementary Material).

**Table 1.** The average terminal angular speeds of spin, rotation and revolution of GNP dimer of *a* = 100 nm or 150 nm induced by LH/RH 800-nm Bessel beams of *l* = 0, 1, and 2 with a cone angle of *α* = 10°.

(kHz)		LH			RH		
**Order**	**Radius *a***	**Ω**_**C**_	**Ω**_**P**_	***ω***_**S**_	**Ω**_**C**_	_**ΩP**_	***ω***_**S**_
*l* = 0	100 nm	-0.05	-0.05	-16.87, -16.86	0.05	0.05	16.87, 16.86
	150 nm	0.19	0.19	-8.46, -8.40	-0.19	-0.19	8.46, 8.40
*l* = 1	100 nm	19.92	-1.21	-32.90	19.91	19.91	35.73, 32.66
	150 nm	28.06	28.06	-18.64, -15.50	15.49	-13.98	17.15
*l* = 2	100 nm	10.18	-2.75	-23.06	11.47	11.47	26.00, 22.00
	150 nm	12.75	12.75	-13.03, -10.25	7.89	-7.55	11.69

**Fig. 3:**
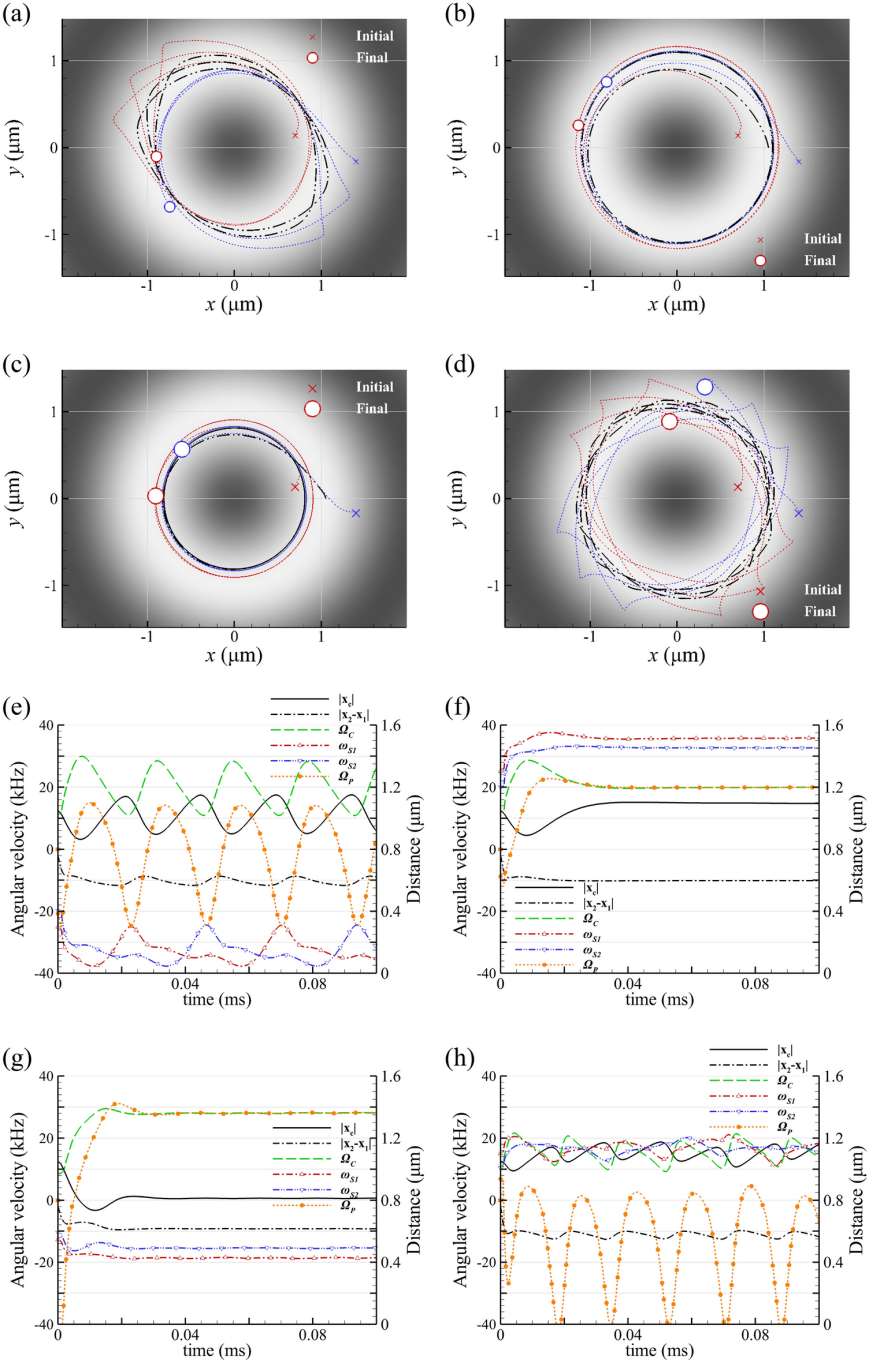
The trajectories of a smaller GNP dimer of *a* = 100 nm, irradiated by a (**a**) LH and (**b**) RH 800-nm Bessel beam of *l* = 1 with a cone angle *α* = 10°. The trajectories of a larger GNP dimer of *a* = 150 nm, irradiated by a (**c**) LH and (**d**) RH Bessel beam of *l* = 1; the blue and red lines: trajectories of two GNPs, and black line: trajectory of their COM. (**e**) to (**h**) The orbital radius of COM and the angular speeds of spin, rotation and revolution of GNP dimer of *a* = 100 nm or 150 nm versus time, corresponding to (**a**) to (**d**), respectively. The periods of the variation in (e) are 0.0237 ms (orbital radius, rotation and revolution), and 0.0474 ms (spin), respectively. The periods of the variation in (**h**) are 0.0173 ms (orbital radius, rotation and revolution), and 0.0346 ms (spin), respectively. In (**e**)-(**h**), the black solid line: the revolution radius of COM’s trajectory, and the black dash line: the distance between two GNPs.

Figures 3a to 3d show the trajectories of two GNPs of a = 100 nm or 150 nm irradiated by a LH or RH Bessel beams of *l* = 1 with α = 10°. Since the order l (topological charge) is positive, the direction of the orbital revolution of the dimer’s COM is CCW. The corresponding orbital radius of COM and the angular speeds of spin, rotation and revolution of GNP dimer versus time versus time are depicted in Figs. 3e to 3h. For a LH Bessel beam of *l* = 1 irradiating two smaller initially separated GNPs (*a* = 100 nm), the rotational direction of the dimer is CW because of the LH Bessel beam, as shown in Figs. 3a and 3e. All the spins, dimer’s rotation and COM’s orbital revolution display quasi-periodic motions. The average rotation and revolution speeds of this dimer of *a* = 100 nm irradiated by LH Bessel beam are Ω_p_ = -1.21 kHz and Ω_c_ = 19.92 kHz, respectively, as shown in Fig. 3e and Table I. The variations, shown in Figs. 3a and 3e, indicate that there is a precession in the orbital motion. This precession in COM’s orbital revolution is due to the difference between the angular speeds of revolution and rotation, particularly when they are in opposite directions. The periods of the variation in Fig. 3e are 0.0237 ms for Ω_c_, Ω_*p*_ and the orbital radius, and 0.0474 ms for *ω*_s_, respectively; the corresponding frequencies of fluctuation are 42.2 kHz and 21.1 kHz, respectively. For the case of RH Bessel beam of *l* = 1, and *a* = 100 nm, the rotation direction of this smaller dimer is CCW because of the RH Bessel beam. Since the directions of the rotation and revolution are consistent (CCW), this is no precession in the COM’s orbital revolution, resulting in a circle orbit for this dimer’s COM with equal angular speeds for both rotation and revolution, as shown in Figs. 3b and 3f. The dynamic motions of the two GNPs induced by Bessel beam of *l* = 1 are referred to Visualization 2.

For a bigger GNP dimer (*a* = 150 nm) irradiated by a RH Bessel beam of* l *= 1, the reverse rotation of dimer occurs due to the negative optical torque, leading to the inconsistent directions of the rotation (CW) and revolution (CCW), as shown in Figs. 3d and 3h. As a result, a precession in the orbital revolution is observed for this case. Again, the variations of orbits, shown in Figs. 3d and 3h, indicate the precession in the orbital motion. The periods of the variation in Fig. 3h are 0.0173 ms for Ω_c_, Ω_p_ and orbital radius, and 0.0346 ms for *ω*_s_, respectively; the corresponding frequencies of fluctuation are 57.8 kHz and 28.9 kHz, respectively. The average rotation and revolution angular speeds of this dimer of *a* = 150 nm irradiated by RH Bessel beam are -13.98 kHz and 15.49 kHz, respectively (Fig. 3(h) and Table I). This inconsistent behavior of the bigger dimer’s rotation and revolution (Figs. 3d and 3h) is different from that of a smaller one (Figs. 3b and 3f), as both are irradiated by the same RH Bessel beam. On the other hand, Figs. 3c and 3g show that the trajectories of two GNPs are circles (inner and outer circles), as irradiated by LH Bessel beam, optically bound together to form a dimer performing a rigid-body rotation and circling motion. Hence, the directions and the angular speeds of the rotation of dimer’s COM and the orbital revolution are the same; CCW and 28.06 kHz. In contrast, for the case of a LH Bessel beam of *l* = 1, the directions of the rotation and revolution of 150-nm GNP dimer are consistent (CCW); the precession is absent as shown in Figs. 3c and 3g. The rotation direction of this bigger dimer opposes the handedness of Bessel beam, whereas the direction of the orbital revolution is guided by the OAM of a Bessel beam of a positive order. These complicated motions involve the SOI facilitated by light-scattering of the multimode of the two GNPs. Note that all these terminal angular speeds of GNP dimer, as listed in Table I, are linearly proportional to the intensity of Bessel beam. The results of a GNP dimer of *a* = 50 nm irradiated by a LH or RH Bessel beam of *l* = 1 are shown in Figure S3 (Supplementary Material). In summary, the rigid-body rotation of the dimer always exists. In most cases, the speed of the dimer’s rigid-body rotation matches the speed of its orbital revolution, as shown in Fig. 3f and 3g. However, in certain special cases, if precession is present, the two speeds can differ, as shown in Fig. 3e and 3h.

**Fig. 4:**
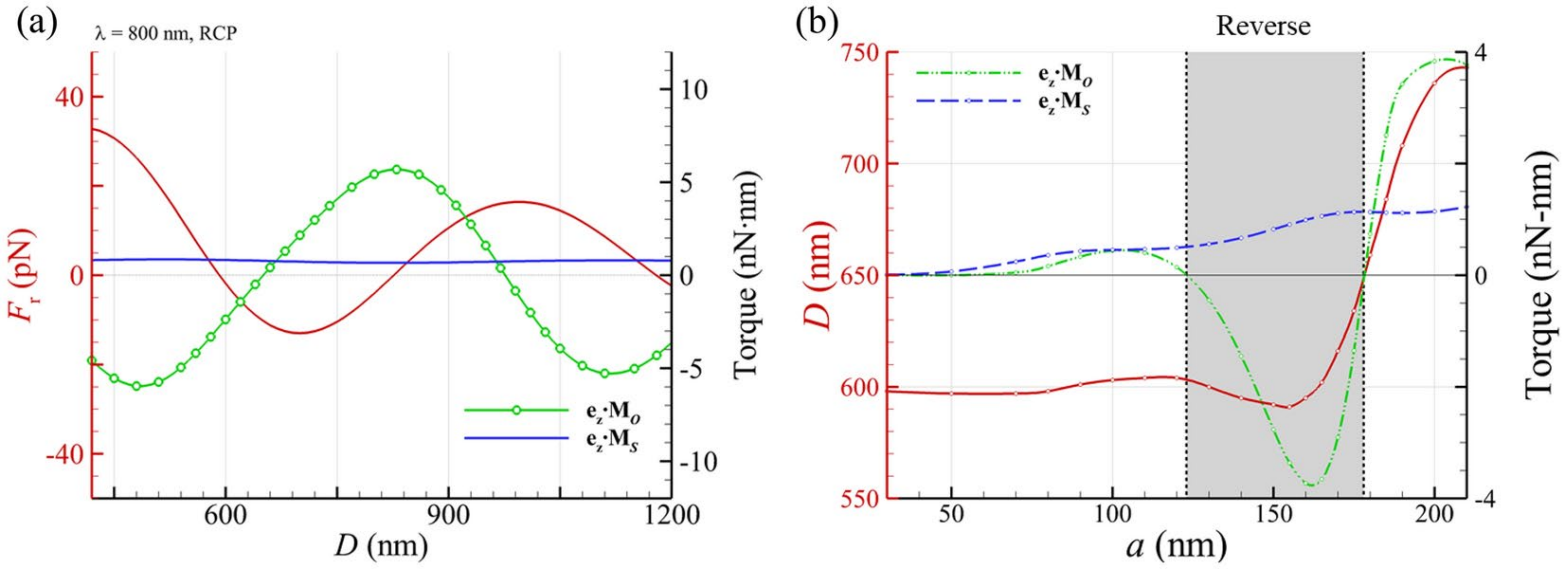
(**a**) Optical force and torque on GNP dimer of* a* = 150 nm irradiated by an 800-nm RCP plane wave versus distance *D* between two GNPs. The first and second stable distances are 592 nm and 1179 nm, respectively; the points with zero cross and a negative slope of *F*_*r*_. (**b**) The first stable distance *D* and the corresponding optical torque (green line) on a GNP dimer of various the radius *a* of GNP. The grey zone indicates the negative optical torque **e**_z_⋅**M**_*O*_, which can induce a reverse rigid-body rotation of dimer *w. r. t.* the handedness of the incident light; the negative optical-torque zone is between *a* = 123 nm and 178 nm.

Figure 4a shows the optical force and torque exerted on two GNPs of* a* = 150 nm with a distance *D* irradiated by an 800-nm right-handed circularly polarized (RCP) plane wave propagating in the *z* direction. The first and second stable distances between them are 592 nm and 1179 nm, respectively, which are the points of zero cross with a negative slope of *F*_*r*_; these values correspond to the integer multiples of the effective wavelength in the medium. Figure 4b shows the size effect of GNP on the optical torque **e**_z_⋅**M**_*O*_ driving the rotation of an optical bound dimer caused by a RCP plane wave. There are multiple turning points in the radius of the GNP for the alternating rotation of the dimer caused by the negative optical torque. There are two turning points of the GNP’s radius for the forward/reverse rotation, *a*= 122 nm and 179 nm. The negative optical torque upon a bigger plasmonic dimer provided by a RCP plane wave has been studied^19^. This phenomenon of the reverse rotation of dimer *w. r. t.* the handedness of light could be related to SOI. In contrast, the optical spin torque **e**_z_⋅**M**_*S*_ is always positive for various size, induced by a RCP plane wave.

**Fig. 5:**
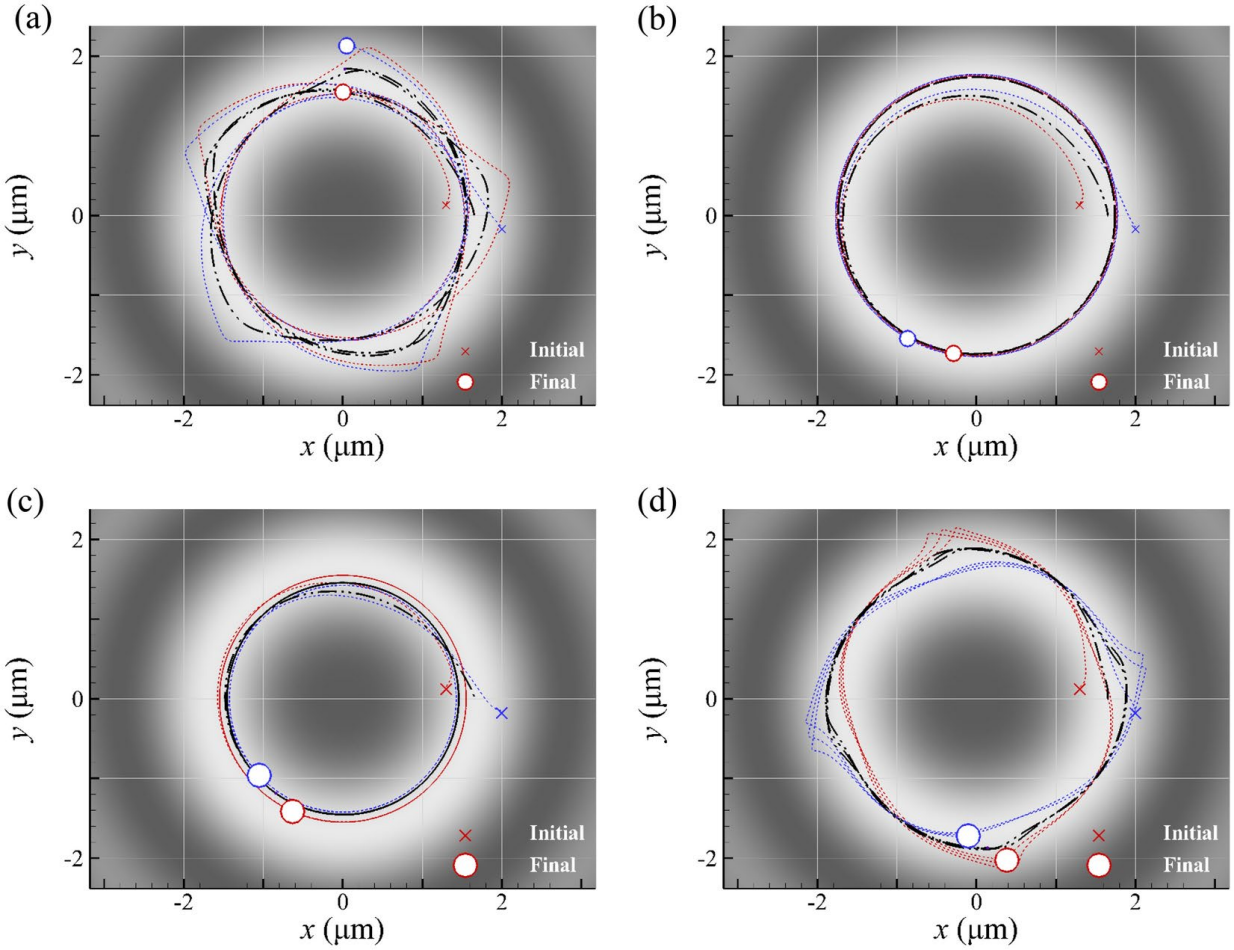
The trajectories of two GNPs of *a* = 100 nm and the COM, irradiated by a (**a**) LH and (**b**) RH Bessel beam of *l* = 2, where *λ* = 800 nm and *α* = 10°. The motions of two GNPs of *a* = 150 nm and the COM, irradiated by a (**c**) LH and (**d**) RH Bessel beam of *l* = 2. The blue and red lines: trajectories of two GNPs, and the black line: trajectory of their COM.

The trajectories of a GNP dimer’s COM, manipulated by a LH or RH Bessel beam of *l* = 2 are shown in Fig. 5. The corresponding orbital radius of COM and the angular speeds of spin, rotation and revolution of GNP dimer versus time are shown in Figure S4 (Supplementary Material), and the terminal values are listed in Table I. No matter the initial positions of the two separate GNPs are, they will eventually come together to form a dimer by the optical binding and perform quasi-rigid-body motions. In addition, the COM moves in an orbital trajectory. For the cases of a smaller dimer (*a* = 100 nm) irradiated by a LH Bessel beam of *l* = 2 or a larger dimer (*a* = 150 nm) by a RH one, the directions of rotation and revolution are opposite, and a precession is induced, as shown in Figs. 5a and 5d. In contrast, for the cases of a smaller dimer (*a* = 100 nm) irradiated by a RH Bessel beam or a larger dimer (*a* = 150 nm) by a LH one, the directions of the rotation and revolution are the same. Therefore, there is no precession, as shown in Figs. 5b and 5c; the trajectories of the two GNPs are circles. The terminal angular speeds of the rotation and revolution of GNP dimer are the same constants, as shown in Figure S4 (Supplementary Material) and Table I. Again, the reverse rigid-body rotation is observed for larger GNPs (*a* = 150 nm), caused by the negative optical torque. This size-dependent behavior of dimer’s rotation induced by a Bessel beam of *l* = 2 is similar to that by a Bessel beam of *l* = 1, shown in Fig. 3. We found that these rigid-body motions of a larger plasmonic dimer with a precession induced by a Bessel beam are more complicated than those observed by Refs^5,28^..

Note that precession occurs due to the difference between the angular speeds of revolution and rotation. For example, we use a RH Bessel beam of l = 1 with a smaller cone angle of *α* = 5° to irradiate two GNPs with radii of 100 nm. There is no precession because the angular speeds of revolution and rotation are the same. However, when a LH Bessel beam of *l* = 1 with a cone angle of *α* = 5° irradiates two GNPs with radii of 150 nm, precession is observed, despite the orbital and rotational directions being the same. Both results are shown in Figure S5 (Supplementary Material). We also investigate the effect of Brownian motion on the orbital motion of a single GNP of *a* = 150 nm, which is manipulated by a lower-intensity RH Bessel beam with *l* = 1, as shown in Figure S6 (Supplementary Material). The temperature effect on the random force exerted by water molecules on the GNP due to plasmonic heating is also taken into account. Due to random forces, fluctuations in the orbital motion are observed, but the orbit remains clearly distinguishable. Even for light with a lower amplitude of *E*_0_ = 0.84 MV/m (0.125 MW/cm^2^), the gradient force of the Bessel beam can still confine the GNP within the first ring, guiding its orbital motion and overcoming Brownian motion, while the phase gradient drives its revolution around the beam axis.

## Conclusion

The optomechanical motions of an optically bound GNP dimer manipulated by Bessel beams of different orders were studied. We calculated the equation of motion of two individual GNPs in terms of the optical force and drag fore of fluid to analyze their trajectories. Our results demonstrate that they will come together eventually to form a dimer with a stable distance due to the optical binding between the two GNPs, regardless of their initial positions. Since a Bessel beam has OAM and SAM simultaneously, a complicated rigid-body motions of the GNP dimer are observed, including GNPs’ spin, dimer’s rotation and COM’s orbital revolution. The directions of the spin and the orbital revolution depend on the handedness and the sign of the order (topological charge) of Bessel beam, respectively. Nevertheless, the rotation direction of the dimer *w. r. t.* COM varies with the size of GNPs. In the case of a smaller dimer (e.g. *a* = 100 nm), the direction of dimer’s rotation is consistent with the handedness of the incident light. In contrast, the rotational direction of a larger dimer (*a*= 150 nm) is opposite to that of the incident light’s handedness. This is because the negative optical torque upon a bigger GNP dimer is induced by CPL^19^. The phenomenon could be due to the SOI induced by the multimode of bigger GNPs. There are multiple turning points in the radius of the GNP for the alternating rotation. Furthermore, we found that a precession may be induced in COM’s orbital revolution as the directions of the rotation and the revolution are opposite. Our finding may provide an insight to the gear motions of GNP clusters manipulated by an optical vortex.

## Supplementary Information

Supplementary Information 1.

Supplementary Video 1.

Supplementary Video 2.

## Data Availability

The datasets generated and/or analyzed during the current study can be obtained on request from the corresponding author.

## Abbreviations

COM: Center of Mass
CPL: Circularly Polarized Light
EM: Electromagnetic
GNP: Gold Nanoparticle
LCP: Left-handed Circularly Polarized Light
LH: Left-Handed
LPL: Linearly Polarized Light
MMP: Multiple Multipole
OAM: Orbital Angular Momentum
RCP: Right-handed Circularly Polarized Light
RH: Right-Handed
SAM: Spin Angular Momentum
SNP: Silver Nanoparticle
SOI: Spin-Orbit Interaction
