# Supplementary material for: Optomechanical motions of gold dimer’s spin, rotation and revolution manipulated by bessel beam
Source: Sci Rep. 2024 Nov 4;14:26714. doi: 10.1038/s41598-024-77413-7 (PMC11535330; doi:10.1038/s41598-024-77413-7)
Supplement: Supplementary file 1 — Supplementary Information 1. [file 41598_2024_77413_MOESM1_ESM.docx]

**Supplementary Material**

**Optomechanical Motions of Gold Dimer’s Spin, Rotation and Revolution Manipulated by Bessel Beam**

Chao-Kang Liu^1^, Yun-Cheng Ku^1,2^, Mao-Kuen Kuo^1*^, Jiunn-Woei Liaw^2,3,4*^

^1^Institute of Applied Mechanics, National Taiwan University, Taiwan

^2^Department of Mechanical Engineering, Chang Gung University, Taiwan

^3^Department of Mechanical Engineering, Ming Chi University of Technology, Taiwan

^4^Proton and Radiation Therapy Center, Linkou Chang Gung Memorial Hospital, Taiwan

Corresponding: [mkkuo@ntu.edu.tw](mailto:mkkuo@ntu.edu.tw), [markliaw@mail.cgu.edu.tw](mailto:markliaw@mail.cgu.edu.tw)

The motions of a pair of identical gold nanoparticles (GNPs) manipulated by left-handed (LH) or right-handed (RH) Bessel beams of different orders are studied. The surrounding medium is water (refractive index *n*= 1.33). Figure S1 shows the normalized intensity distributions of different-order Bessel beams of *l*= 0, 1 or 2, where the wavelength *λ* is 800 nm and the cone angle *α* is 10°. The radii of the first peak of Bessel beams of *l*= 0, 1 or 2 are 2115 nm, 1020 nm and 1690 nm, respectively.


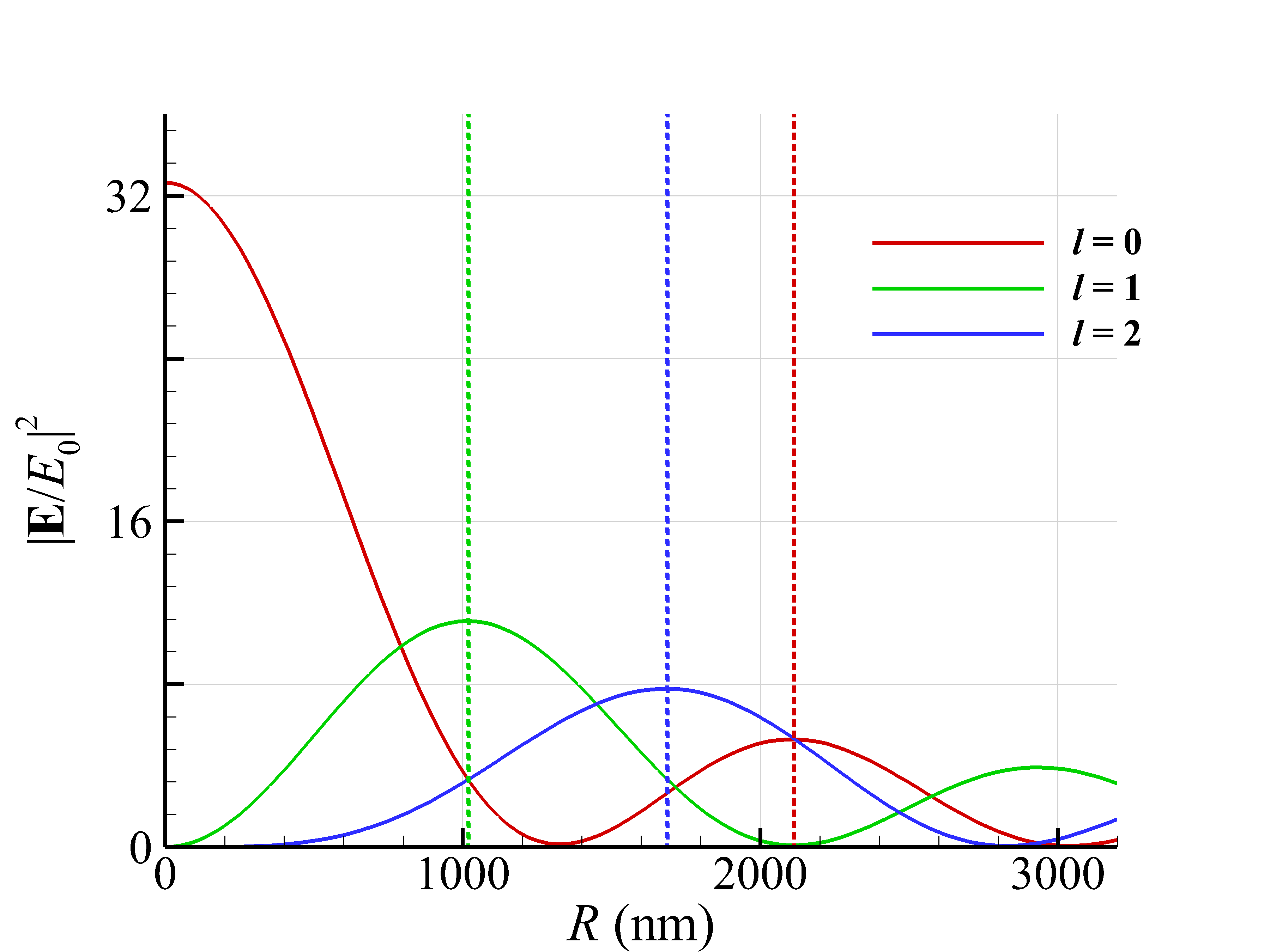


(b)

**Figure S1.** The intensity distributions of 800-nm Bessel beams of *l*= 0, 1 and 2 with a cone angle of *α =* 10°.

The motions of the two individual GNPs in water are analyzed. Figure S2a shows the trajectories of two GNPs of *a*= 50 nm and their center of mass (COM) under the irradiation of a RH Bessel beam of *l*= 0, propagating along *z* direction. The amplitude of Bessel beam is $E_{0}=\text{11.89 (}\frac{\text{MV}}{\text{m}}\text{)}$. No matter the initial positions of the two GNPs are, they will be optically bound together to form a dimer eventually with a distance of about 600 nm between them, close to the effective wavelength of light in water. Afterward, the two GNPs are trapped within the intensity peak of a Bessel beam ring, where they remain nearly stationary. Nevertheless, both GNPs undergo spinning. The angular speeds of spin, rotation and revolution of the two GNPs versus time are plotted in Figure S2b. This phenomenon is because Bessel beam of *l*= 0 only has spin angular momentum (SAM), but does not have orbital angular momentum (OAM) intrinsically.


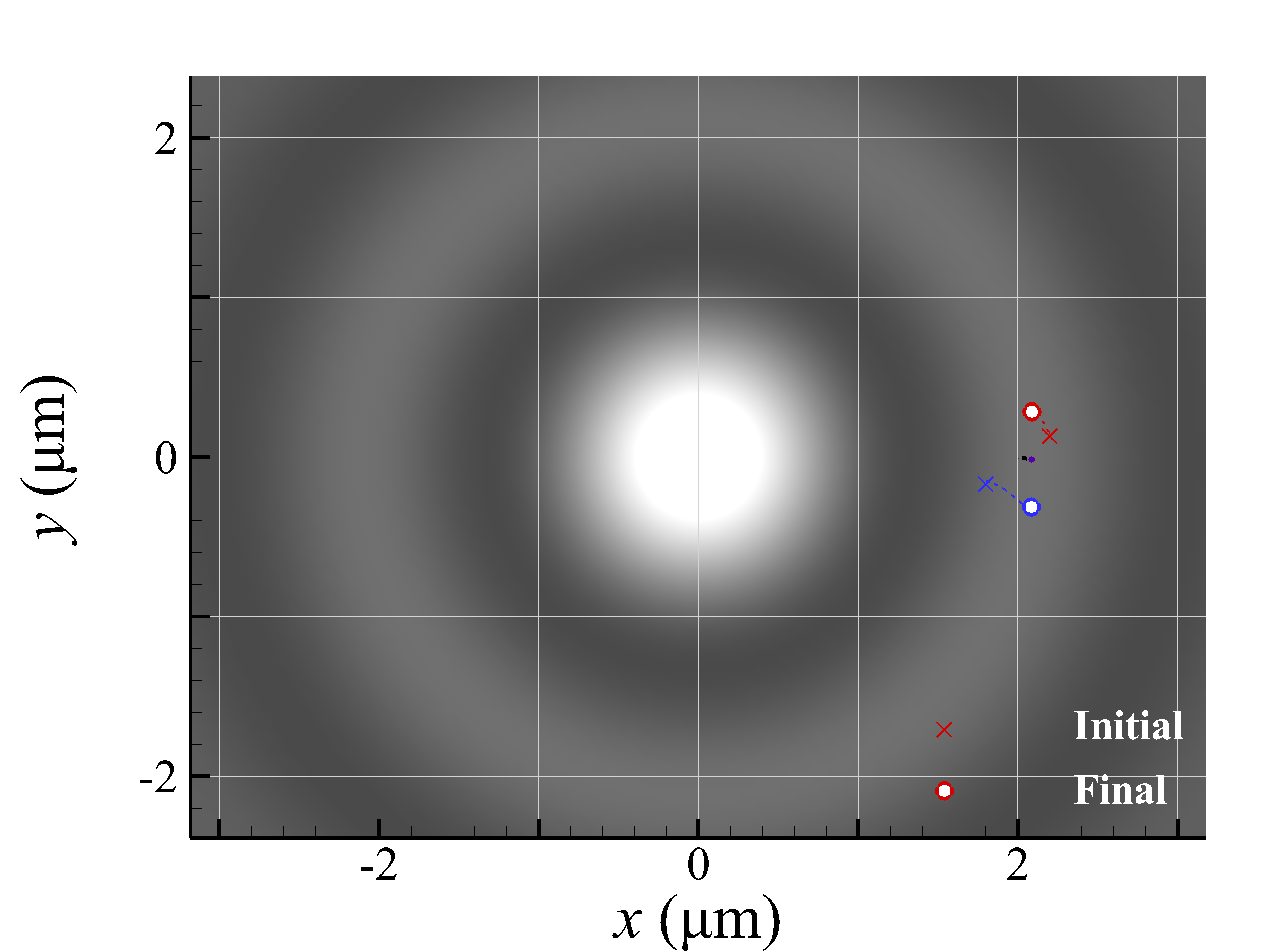


(a)


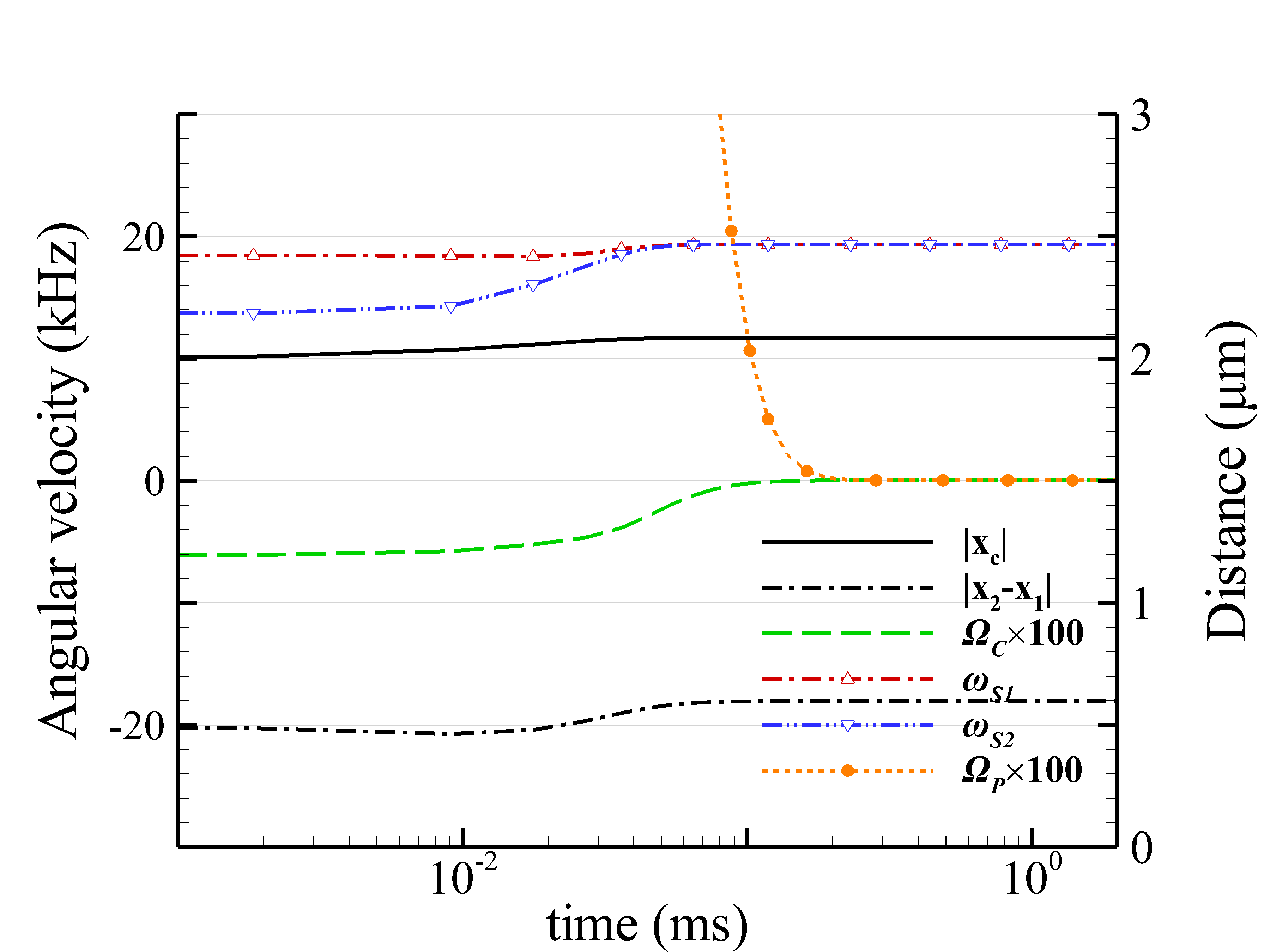


(b)

**Figure S2.** (a) Trajectories of two GNPs of *a*= 50 nm irradiated by 800-nm RH Bessel beam of *l*= 0 and *α =* 10°. (b) The orbital radius of COM and the angular speeds of spin, rotation and revolution of GNP dimer versus time. The black solid line: the radius of COM’s trajectory, and the black dash line: the distance between two GNPs.

Additionally, for smaller GNPs of *a*= 50 nm, the effect of spin-orbit interaction (SOI) via the light scattering is very weak, so there is almost no rigid-body rotation and orbital revolution. In contrast, the SOI effect becomes severe for larger GNPs of *a*= 150 nm, so that a part of SAM of Bessel beam of *l*= 0 is converted into OAM to induce the dimer’s rotation and the COM’s orbital revolution via the light scattering. The average terminal angular speeds of spin, rotation and revolution of a GNP dimer of *a*= 50 nm induced by LH or RH 800-nm Bessel beams of *l*= 0, 1, and 2 are listed in Table SI. The results of a GNP dimer of *a*= 50 nm irradiated by a LH or RH Bessel beam of *l*= 1 are shown in Figure S3. There is no precession in the COM’s orbital revolution, as shown in Figures S3a and S3c.


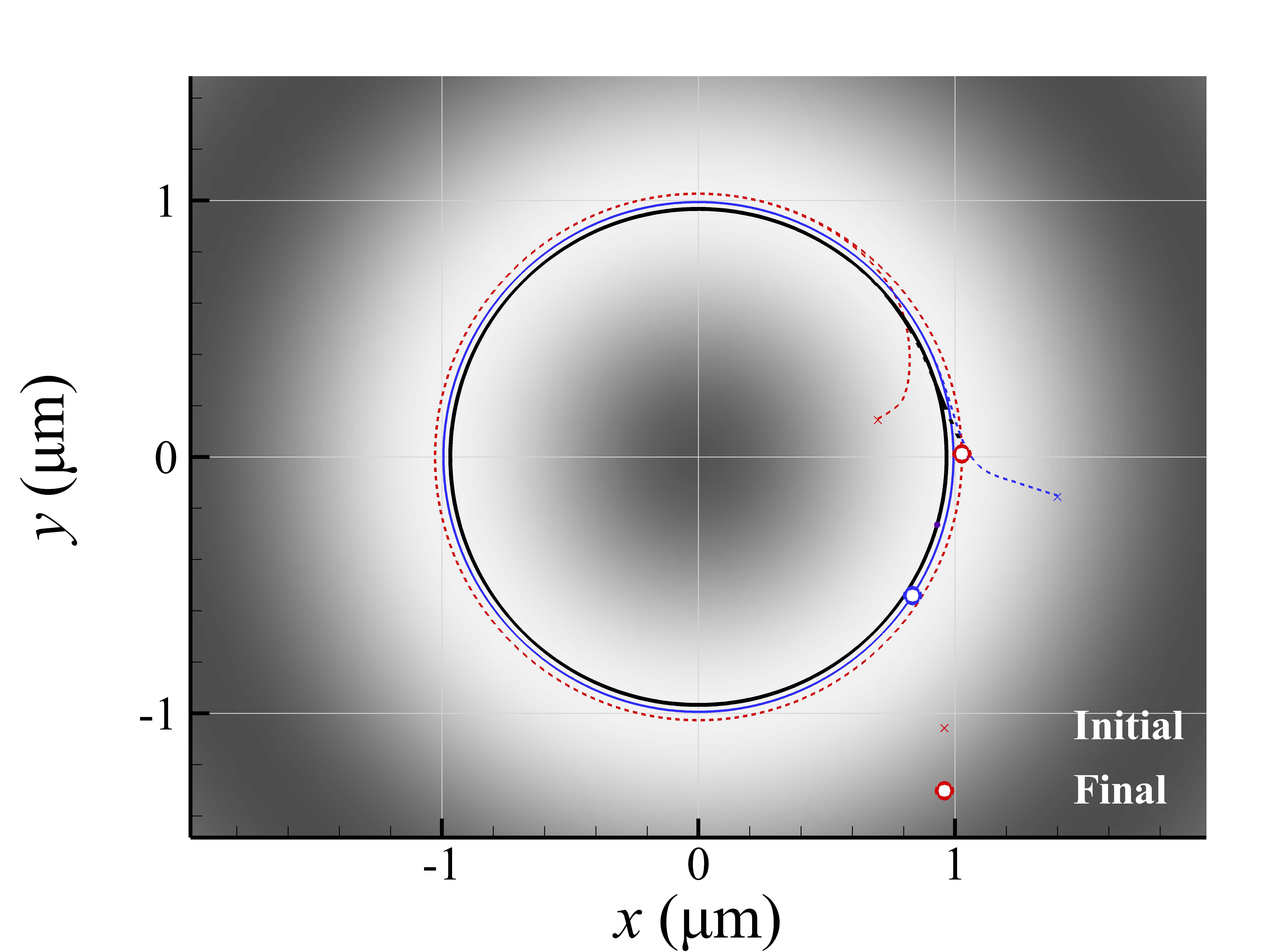


(a)


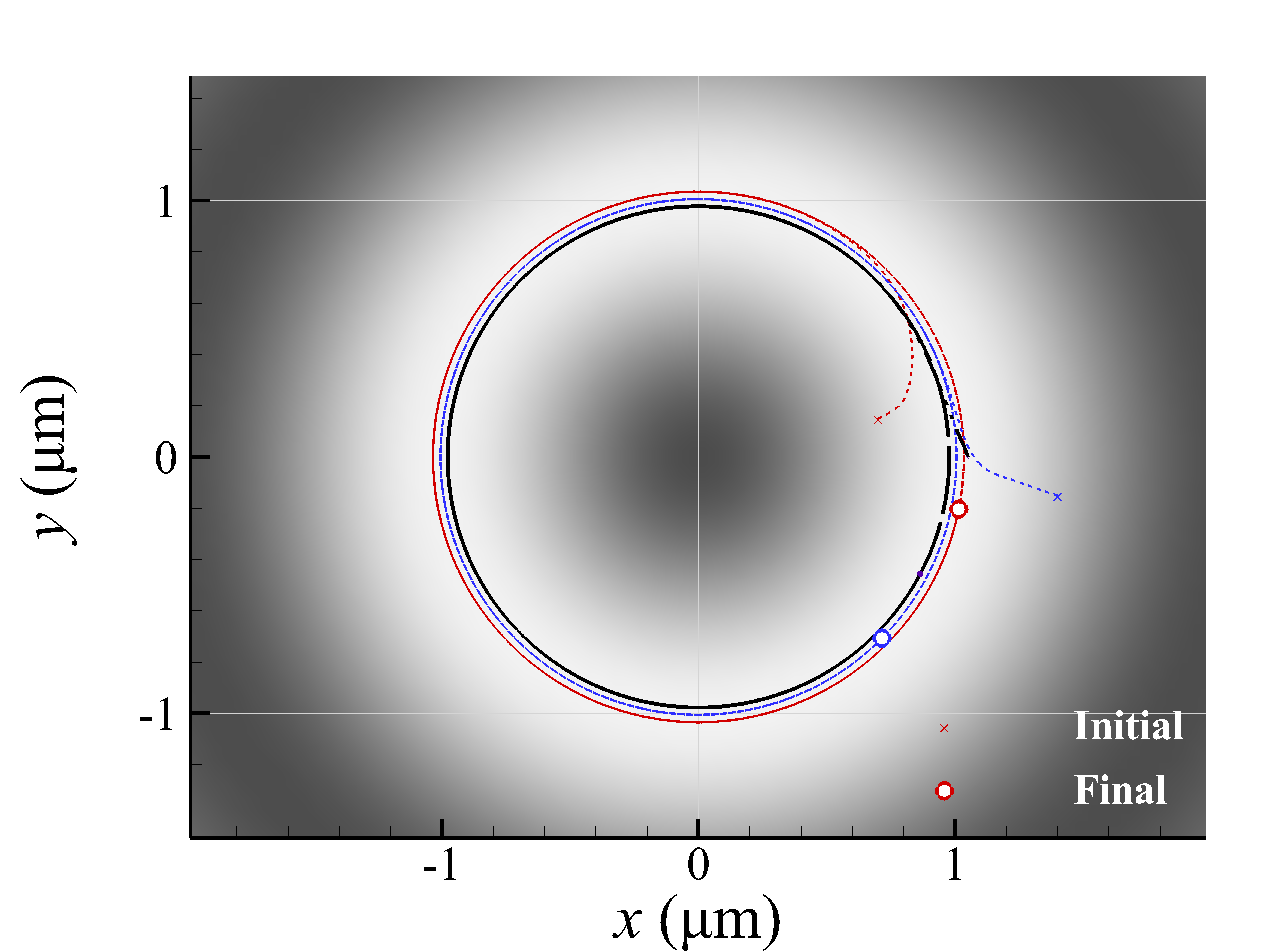


(b)


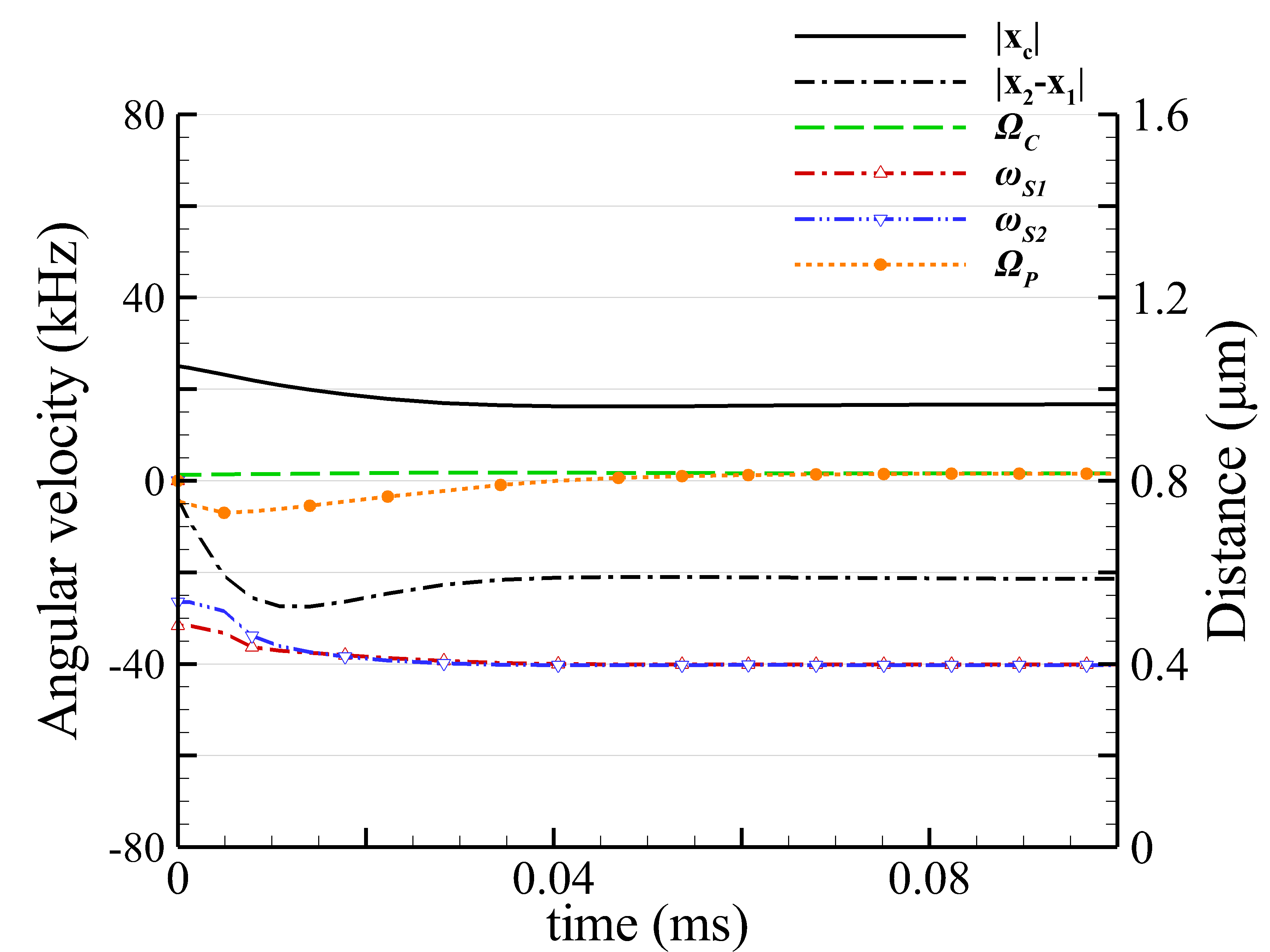


(c)


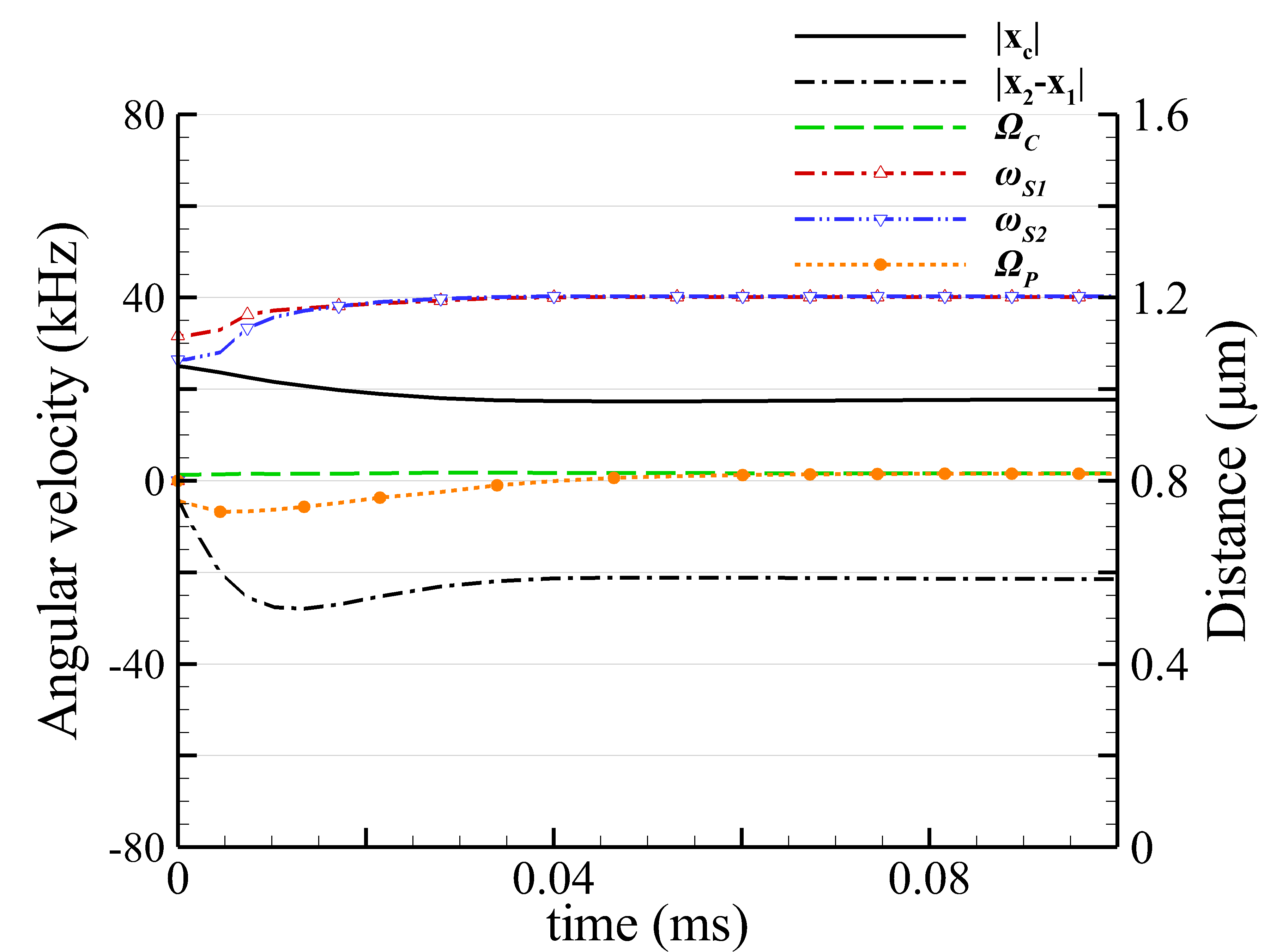


(d)

**Figure S3.** The trajectories of a smaller GNP dimer of *a*= 50 nm, irradiated by a (a) LH and (b) RH 800-nm Bessel beam of *l*= 1 with a cone angle *α =* 10°. (c) and (d) are the orbital radius of COM and the angular speeds of spin, rotation and revolution of GNP dimer versus time, corresponding to (a) and (b), respectively. In (c) and (d), the black solid line: the radius of COM’s trajectory, and the black dash line: the distance between two GNPs.

**Table SI.** The average terminal angular speeds of spin, rotation and revolution of GNP dimer of *a*= 50 nm induced by LH/RH 800-nm Bessel beams of *l*= 0, 1, and 2 with a cone angle of *α =* 10°.

| (kHz) | LH | | | RH | | |
| --- | --- | --- | --- | --- | --- | --- |
| Order | Ω_C_ | Ω_P_ | ω_S_ | Ω_C_ | Ω_P_ | ω_S_ |
| *l*= 0 | -2.55x10^-4^ | -2.55x10^-4^ | -19.35, -19.34 | 2.55x10^-4^ | 2.55x10^-4^ | 19.35, 19.34 |
| *l*= 1 | 1.59 | 1.59 | -40.09, -40.25 | 1.58 | 1.58 | 40.06, 40.29 |
| *l*= 2 | 0.81 | 0.81 | -27.96, -28.11 | 0.81 | 0.81 | 27.94, 28.13 |

The motions of the two individual GNPs of *a*= 100 nm or 150 nm in water under the irradiation of a Bessel beam of *l*= 2 are analyzed. Figure S4 shows the orbital radius of COM and the angular speeds of spin, rotation and revolution of two GNPs of and their COM versus time. The variations in the orbital radius of COM and the angular speeds of the dimer’s rigid-body rotation and COM’s orbital revolution are observed in Figures S4a and S4d, implying a precession in the orbital motion. For the smaller GNP dimer (*a*= 100 nm), the direction of the rotation is CW due to LH Bessel beam, and the direction of the revolution is CCW due to the positive order (*l*= 2), as shown in Figure S4a; they are opposite. For the larger GNP dimer (*a*= 150 nm), the reverse rotation is observed due to the negative optical torque, resulting in the opposite directions of the rotation (CW) and the revolution (CCW), as shown in Figure S4d. For the other cases of Figures S4b and S4c, the angular speeds of rotation and revolution converge towards the same, and the COM’s orbit is a circle eventually. For both cases, there is no precession.


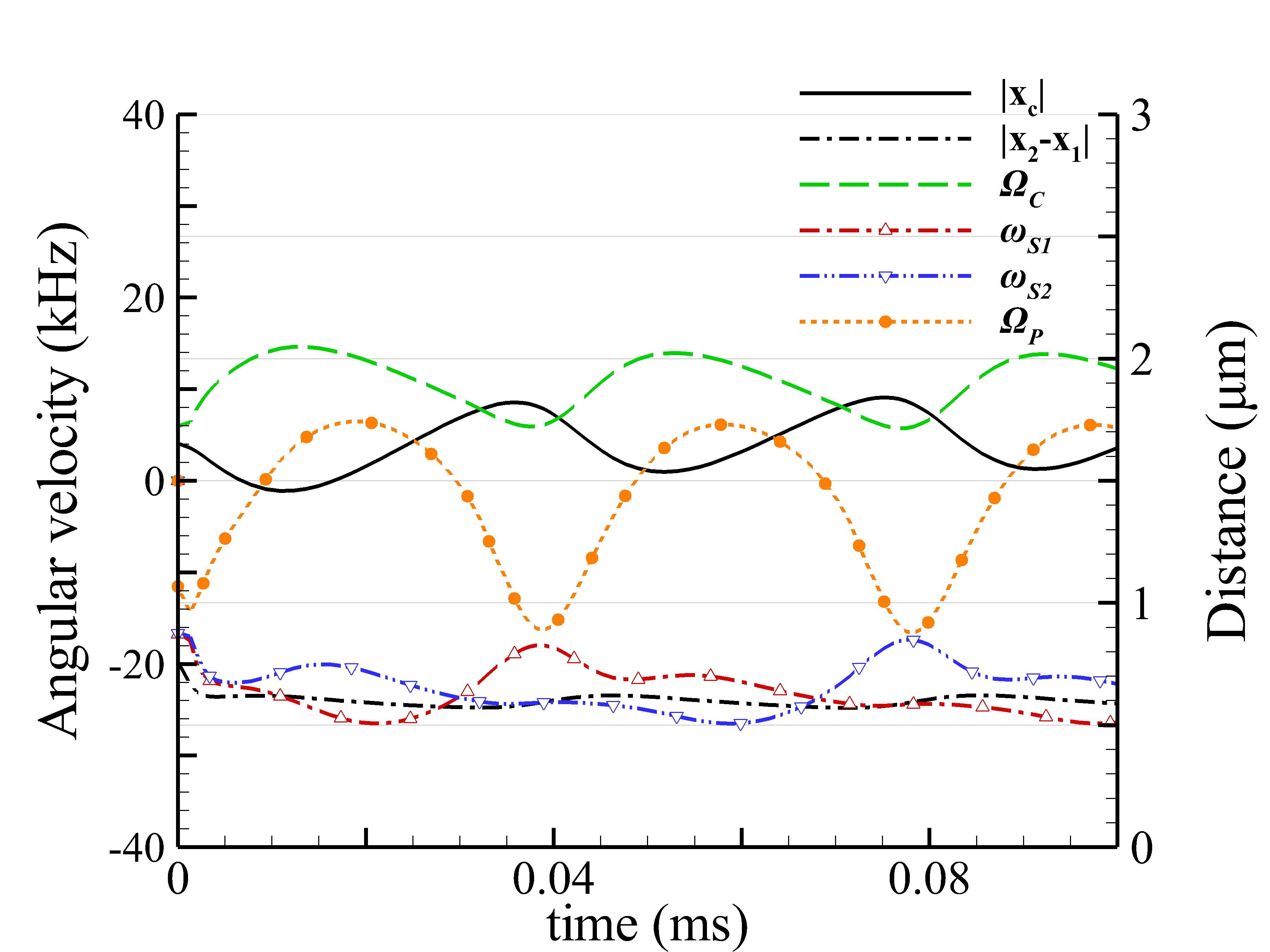


(a)


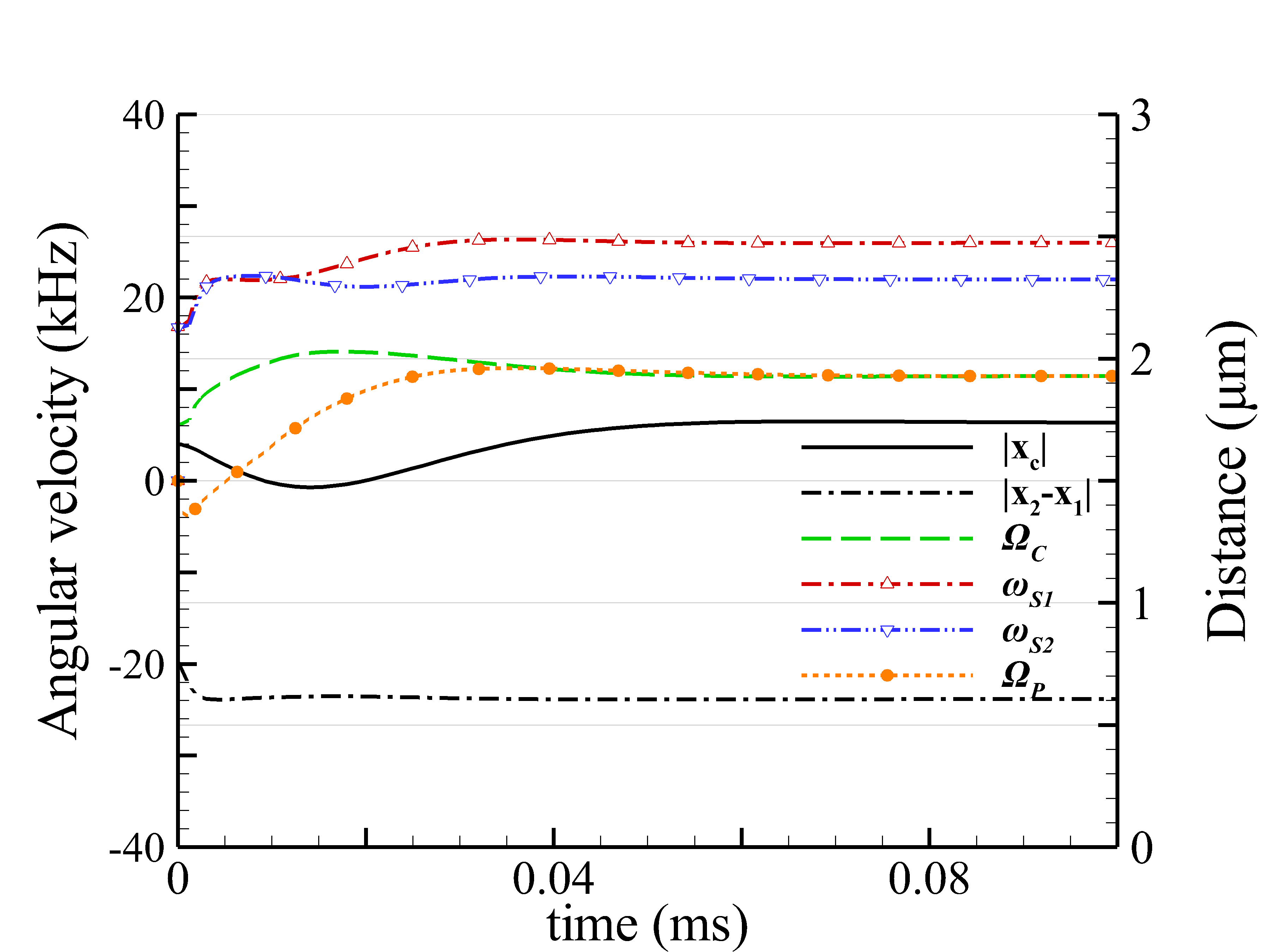


(b)


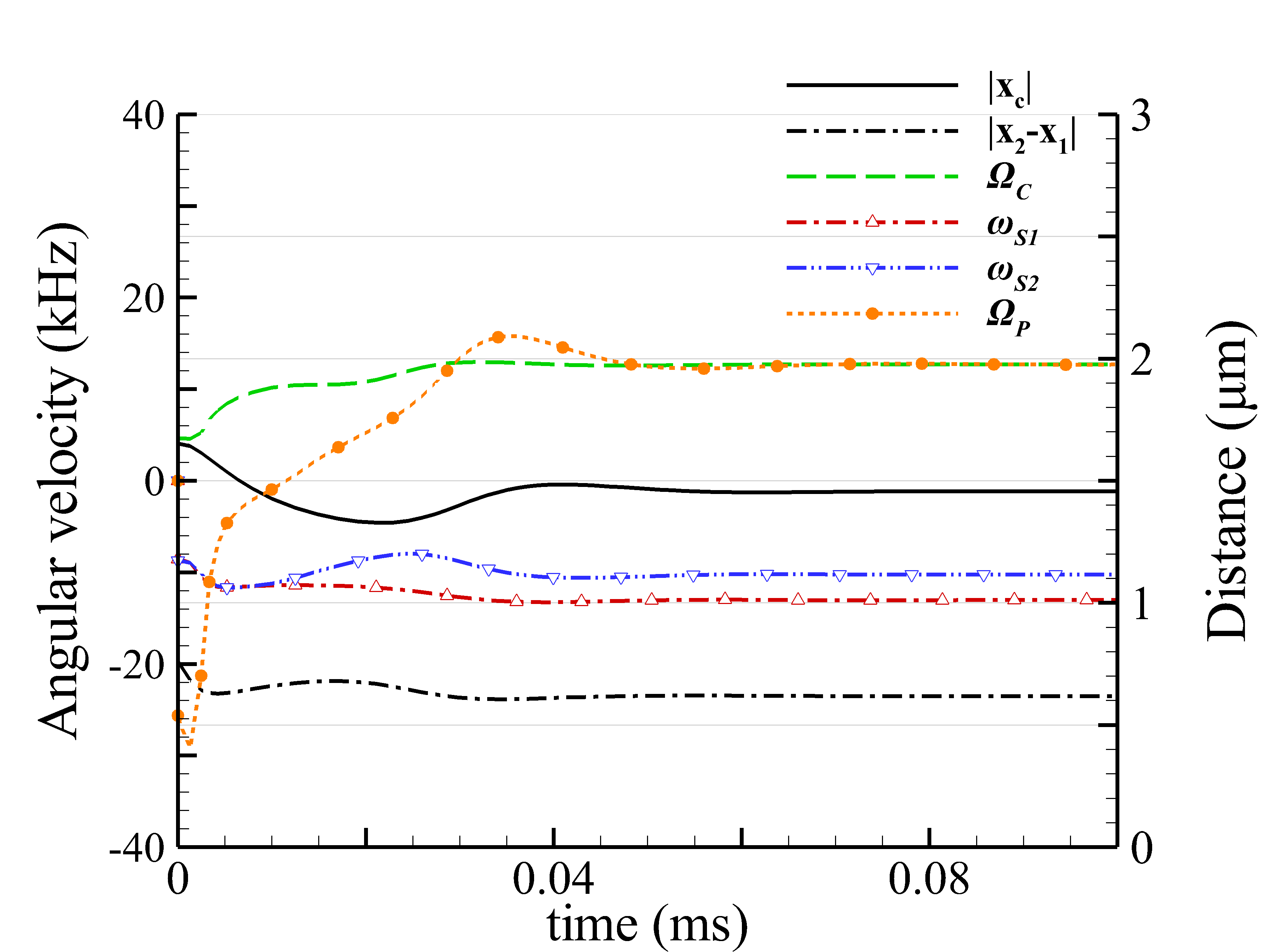


(c)


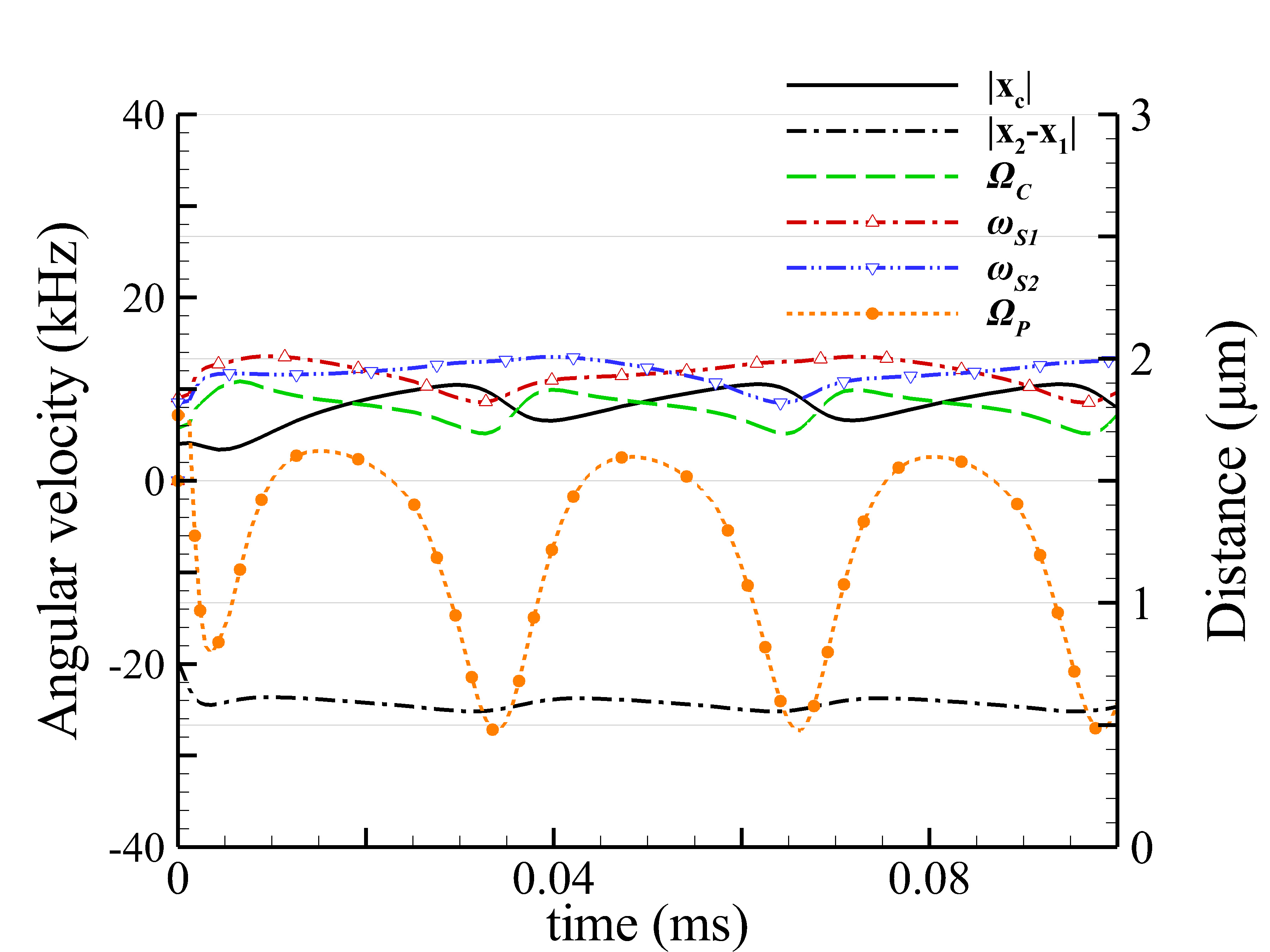


(d)

**Figure S4.** The orbital radius of COM and the angular speeds of spin, rotation and revolution of GNP dimer of *a*= 100 nm, induced by (a) LH and (b) RH Bessel beam of *l*= 2 and *α =* 10°. The results of GNP dimer of *a*= 150 nm induced by (c) LH and (d) RH Bessel beam. Black solid line: orbital radius of COM. The black solid line: the radius of COM’s trajectory, and the black dash line: the distance between two GNPs.

We also study the cone angle effect. For example, Figure S5a shows that result of a RH Bessel beam of *l*= 1 with a smaller cone angle of *α*= 5^o^ irradiating two GNPs with radii of 100 nm. There is no precession because the angular speeds of revolution and rotation are the same, as shown in Figure S5c. However, when a LH Bessel beam of *l*= 1 with a cone angle of *α*= 5^o^ irradiates two GNPs with radii of 150 nm, precession is observed, despite the orbital and rotational directions being the same (Figure S5b, d).


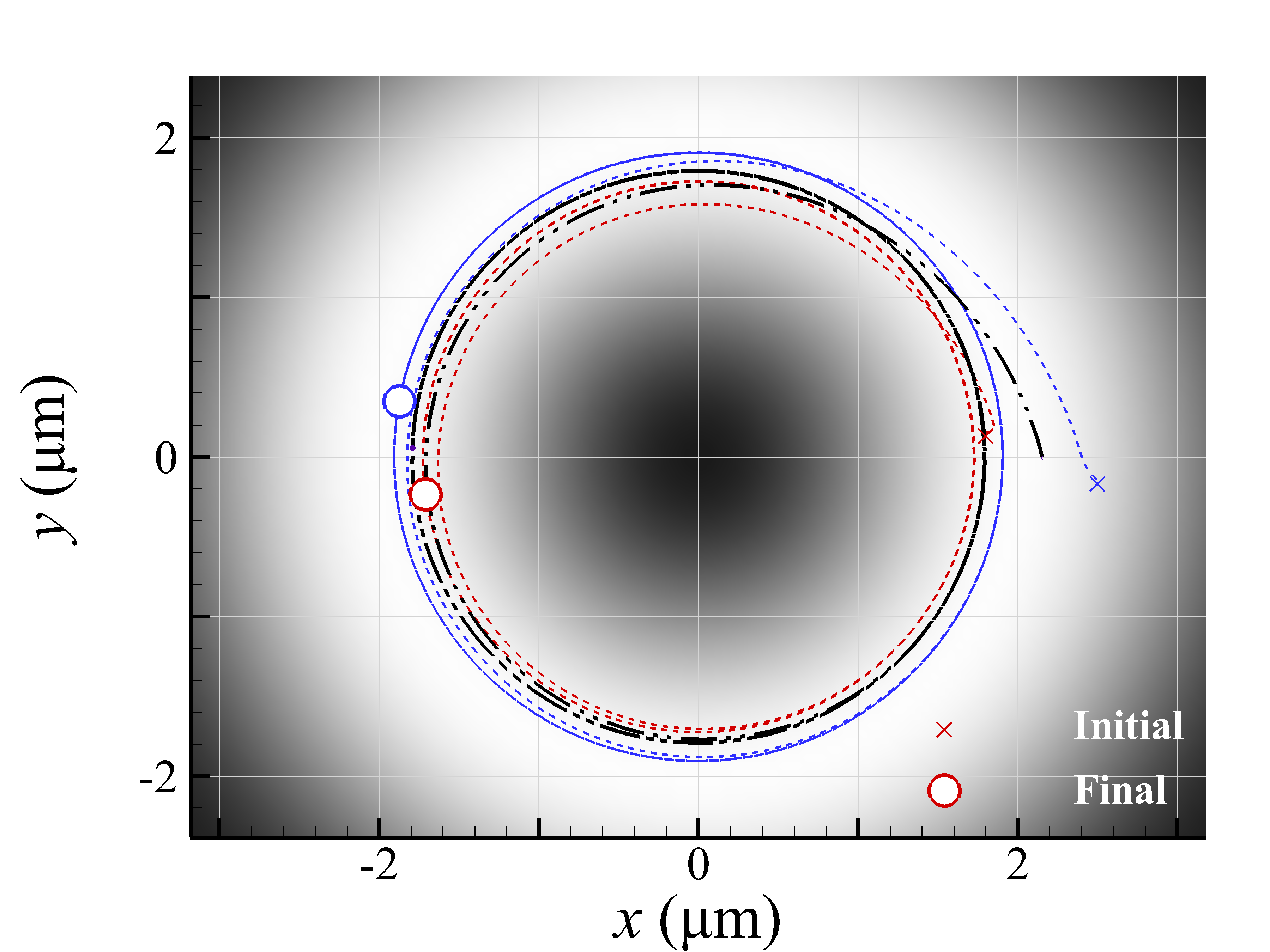


(a)


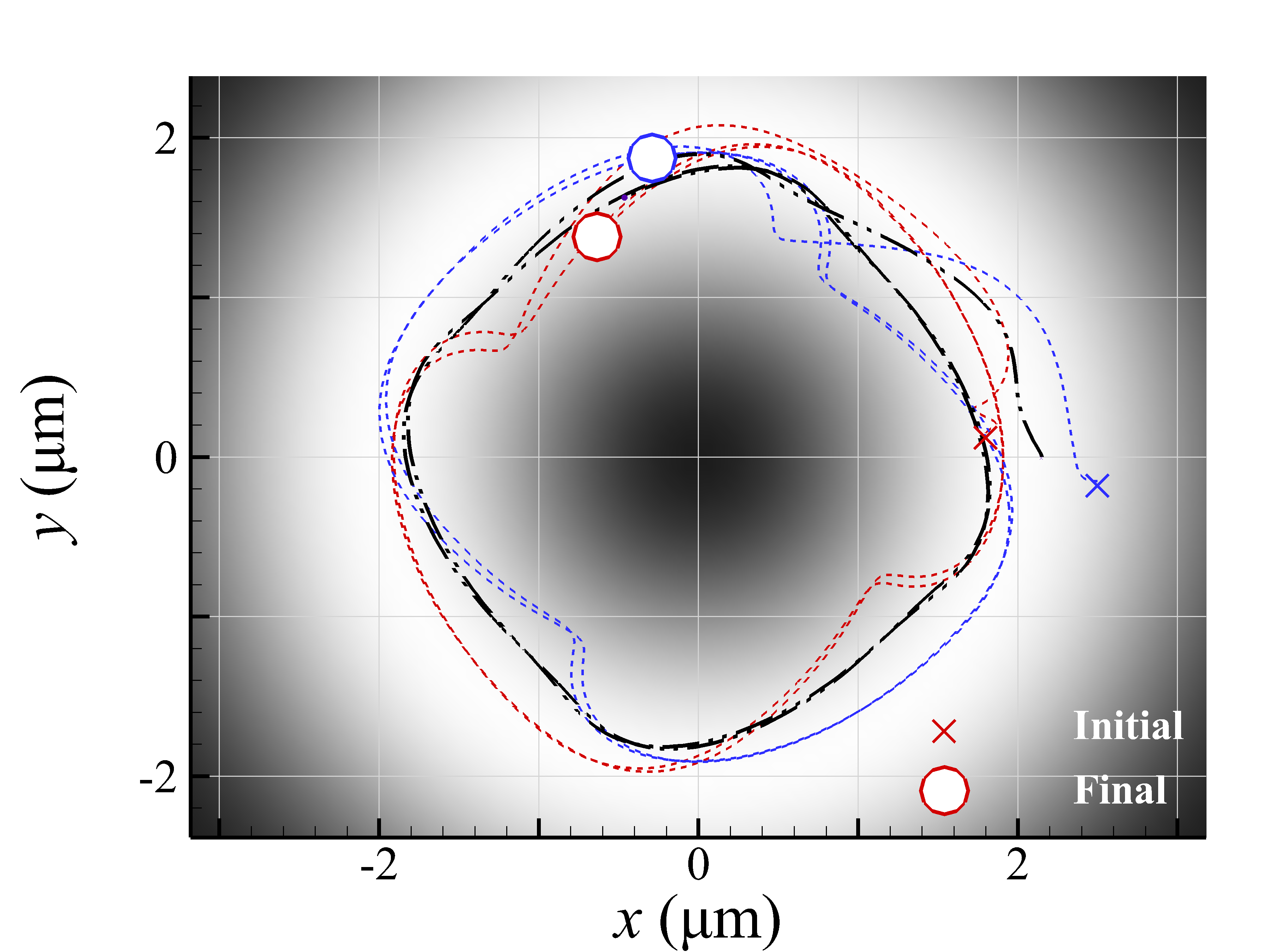


(b)


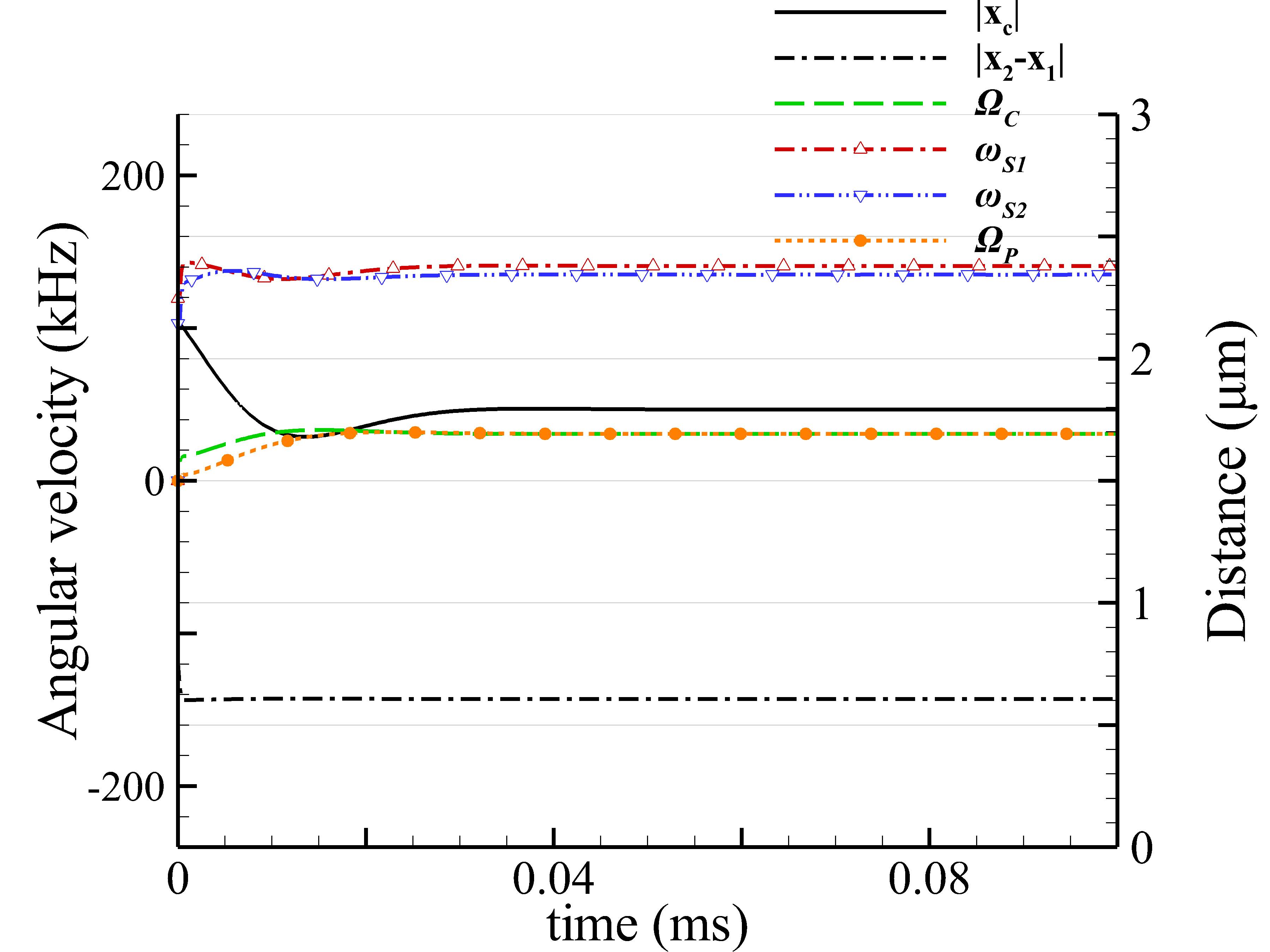


(c)


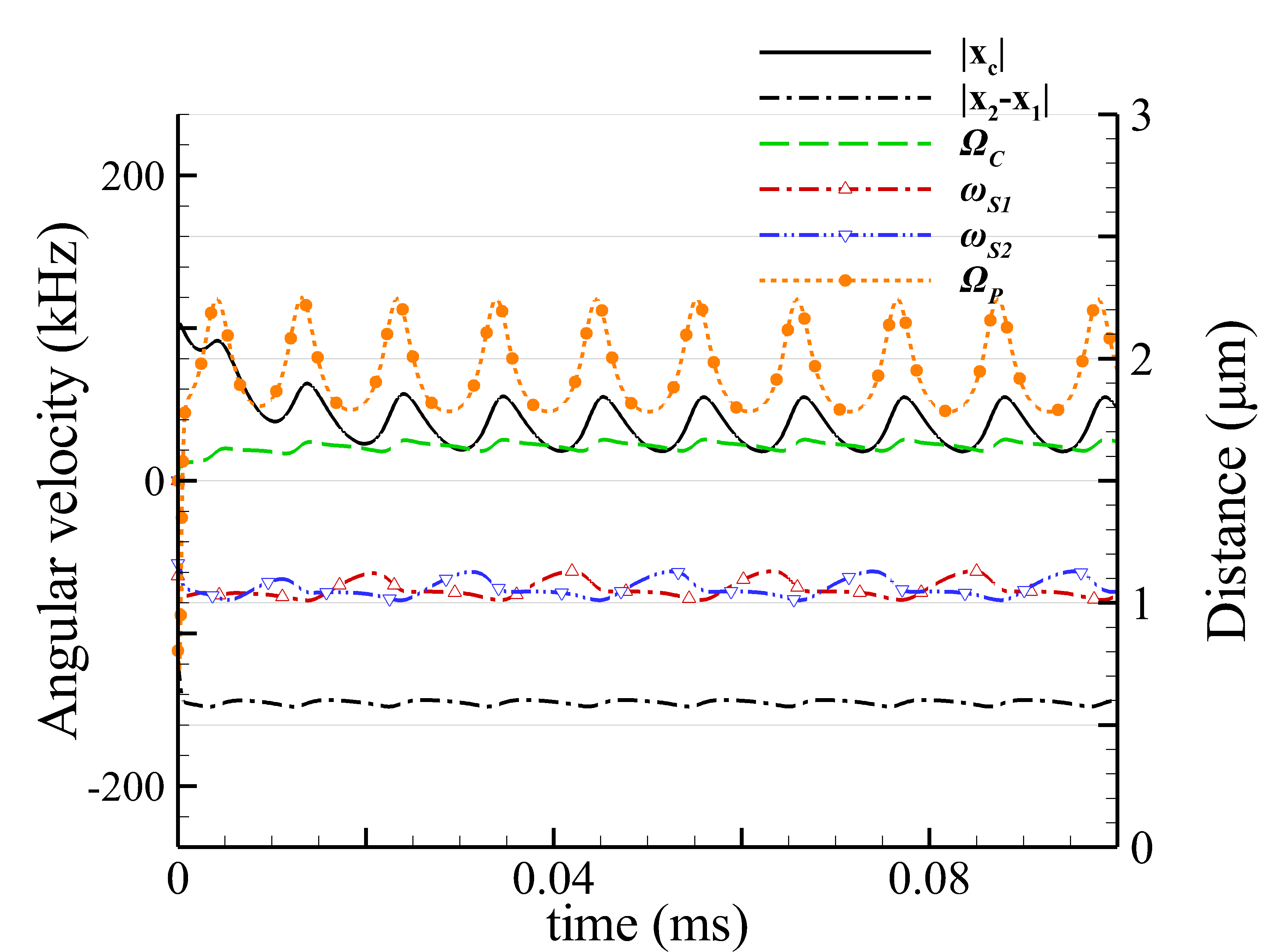


(d)

**Figure S5.** The trajectories of a smaller GNP dimer of (a) *a*= 100 nm, irradiated by a RH and (b) *a*= 150 nm, irradiated by a LH 800-nm Bessel beam of *l*= 1 with a cone angle *α =* 5°. (c) and (d) are the orbital radius of COM and the angular speeds of spin, rotation and revolution of GNP dimer versus time, corresponding to (a) and (b), respectively. In (c) and (d), the black solid line: the radius of COM’s trajectory, and the black dash line: the distance between two GNPs.

We also investigate the effect of Brownian motion on the GNP's movement. To simulate this, a random force is added to the right-hand side of the particle’s equations of motion, as shown below [1].

$m_{i}\frac{d^{2}\mathbf{x}_{i}}{dt^{2}}=-\eta_{i}\frac{d\mathbf{x}_{i}}{dt}+\mathbf{F}_{i}\text{+}\mathbf{F}_{i}^{B}$ (S1)

In Eq. (S1), the random force is expressed as

$\mathbf{F}_{i}^{B}=w\left(t\right)\sqrt{\frac{2k_{B}\left(T_{0}+\Delta T\right)\eta_{i}}{\Delta t}}$ (S2)

Here, *k_B_* is Boltzmann’s constant, and *w*(*t*) represents a sequence of Gaussian random numbers with zero mean and unit variance. If a GNP with a radius *a* of 150 nm is in water, the relaxation time is *τ* = 108.54 ns, the surrounding temperature *T_0_* is 300^o^ K, and the thermal conductivity of water *κ* is 0.6 W/mK. The coefficient *η_i_* is 6π*μ_v_a* with the water viscosity *μ_v_*. When the GNP is irradiated by a Bessel beam, the absorbed power *Q*_abs_​ leads to a temperature increase Δ*T*, which can be expressed in terms of the surface integral of the time-averaged Poynting vector **P***_avg_* ​[2-4].

$\Delta T=\frac{Q_{abs}}{4\pi\kappa a}$ (S3)

$Q_{abs}=\int_{\partial\Omega} -\mathbf{P}_{avg}\cdot\mathbf{e}_{n}dS$ (S4)

$\mathbf{P}_{avg}=\frac{1}{2}\mathrm{Re}\left(\mathbf{E}\times\overline{\mathbf{H}}\right)$ (S5)

We used a lower-intensity RH Bessel beam of *l*= 1 at 800 nm to irradiate a single GNP of *a*= 150 nm to investigate the effect of Brownian motion. When the light intensity is 1.25 MW/cm², the stiffness of the Bessel beam at the first ring, trapping the GNP, is approximately 0.0244 pN/nm. Therefore, we use a time step of Δ*t*= 20 ns in the Runge-Kutta method to simulate the dynamic equation of motion, Eq. (S1).

Figures S6a and S6b show the trajectories of a single GNP at intensities of 1.25 MW/cm² (*E*_0_= 2.66 MV/m) and 0.125 MW/cm² (*E*_0_= 0.84 MV/m), respectively. For both cases, the orbital radius is approximately 1 μm, corresponding to the first ring of the RH Bessel beam with *l*= 1. Figure S6c shows the orbital radius (distance from the optical axis) and angle of GNP versus time for intensity of 1.25 and 0.125 MW/cm^2^. Figure S6d shows the temperature increase Δ*T* and angular speed of GNP versus time. Obviously, the angular speed is proportional to the intensity of light. Due to the random forces exerted by water molecules on the GNP, fluctuations in its orbital motion are observed in both cases. However, the orbital motion remains clearly identifiable. Even at a lower intensity of 0.125 MW/cm², the gradient force of the Bessel beam can still confine the GNP to move along the orbit, overcoming Brownian motion, while the phase gradient drives its rotational motion around the beam axis.


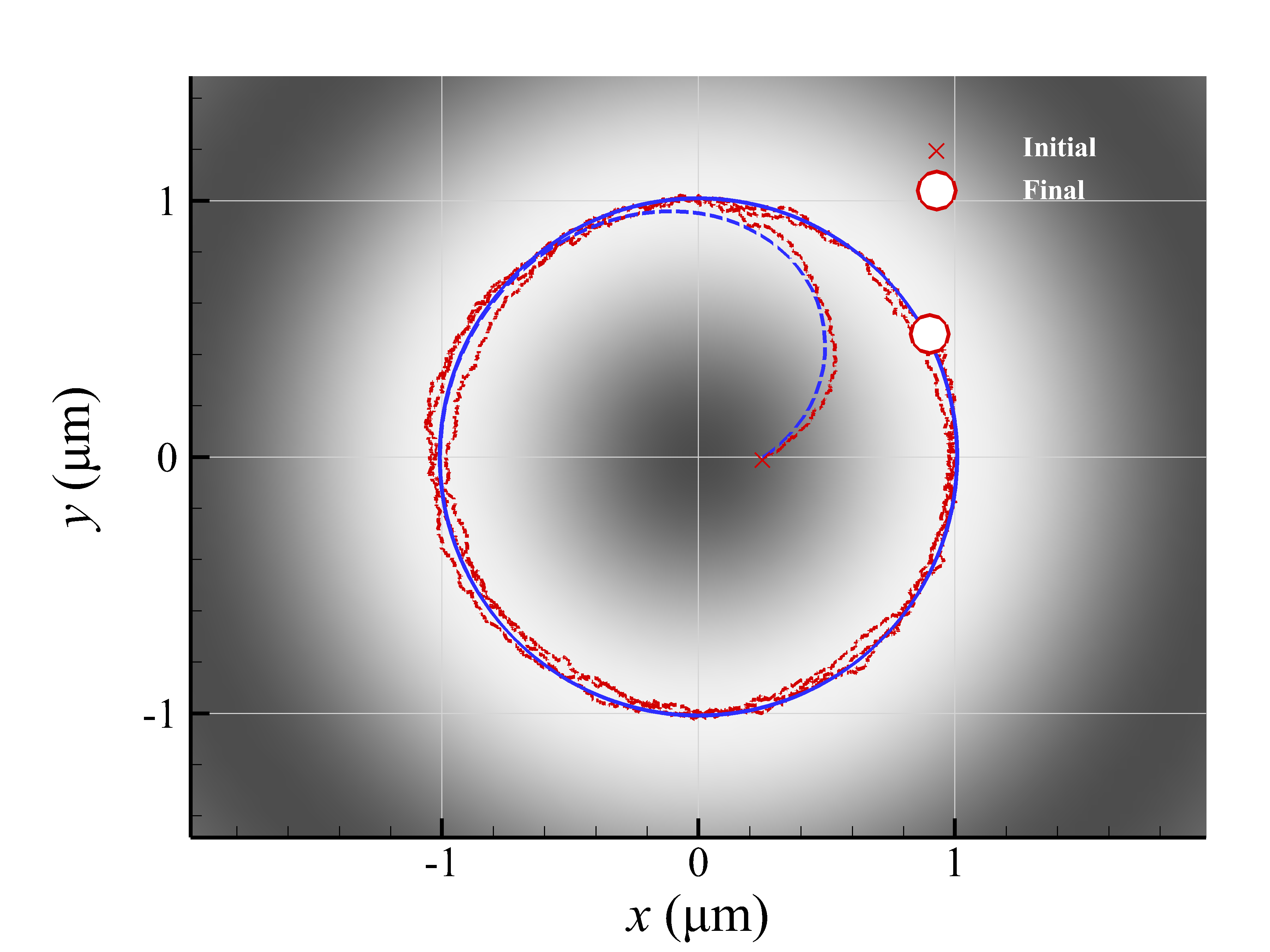


(a)


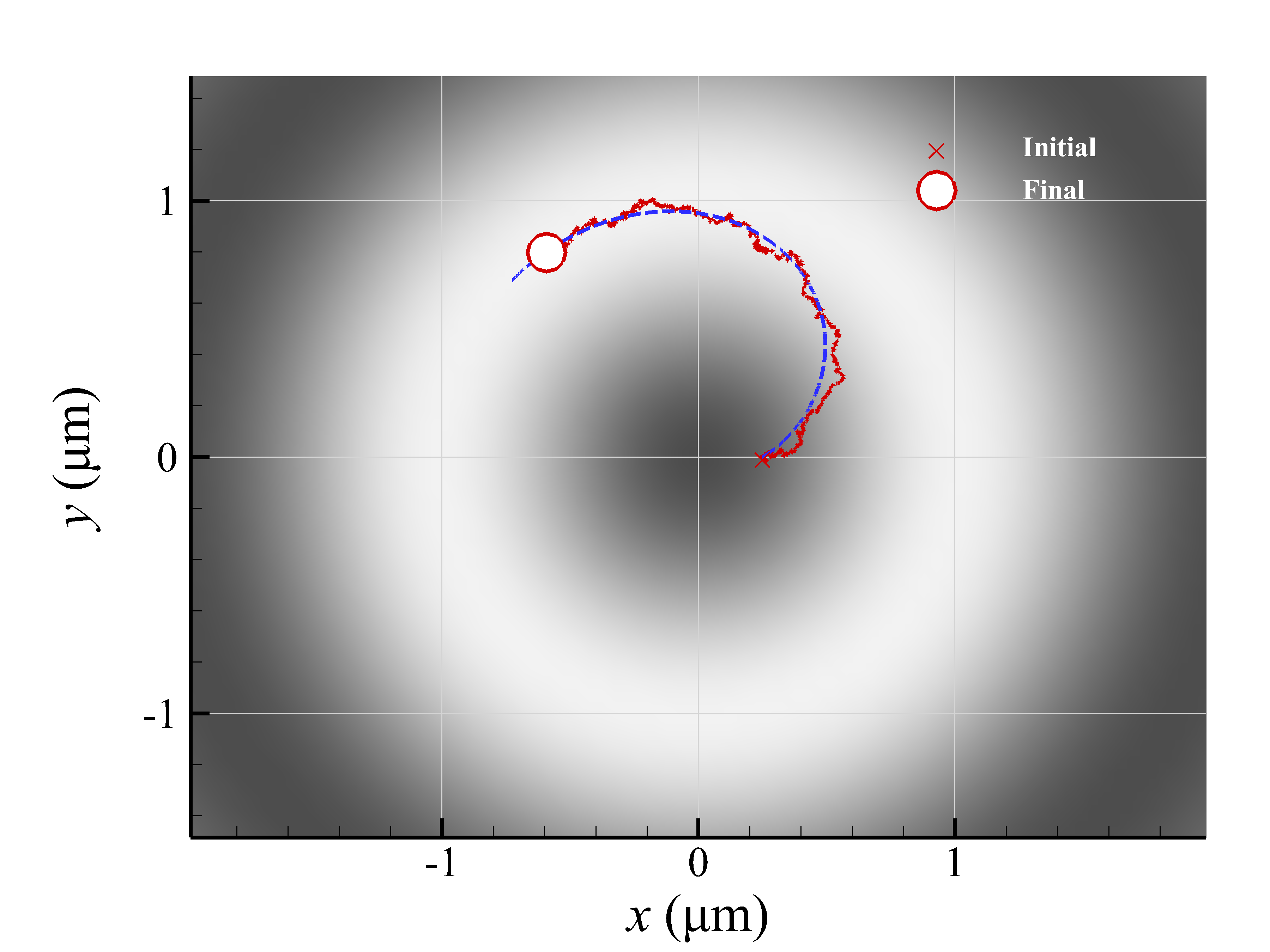


(b)


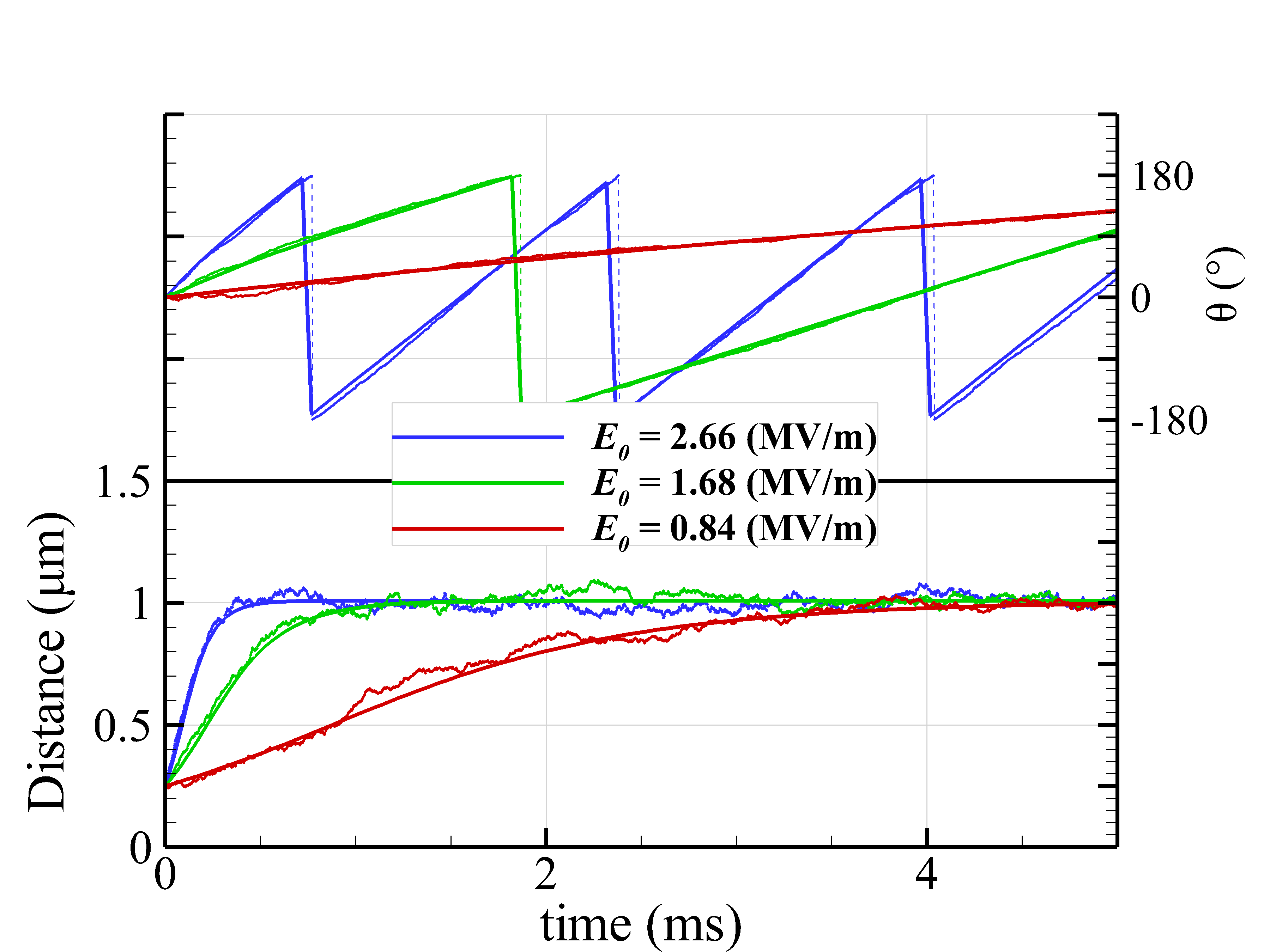


(c)


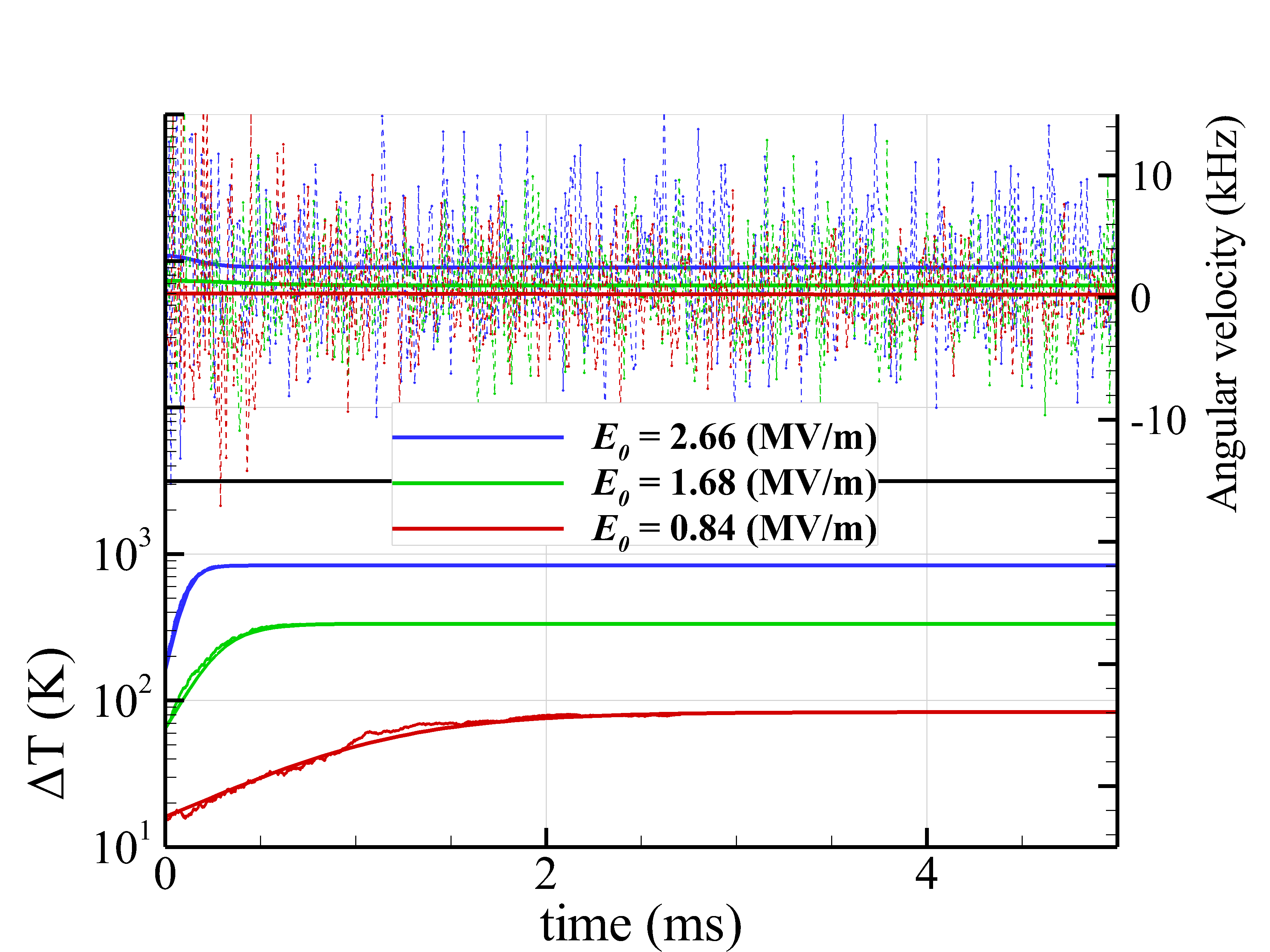


(d)

**Figure S6.** The trajectories of a single GNP of *a*= 150 nm irradiated by a RH 800-nm Bessel beam of *l*= 1 with a cone angle *α =* 10° and (a) light intensity of 1.25 MW/cm^2^ and (b) light intensity of 0.125 MW/cm^2^, respectively. (c) The orbital radius (distance from the optical axis) and angle of GNP versus time for intensity of 1.25 and 0.125 MW/cm^2^. (d) Δ*T* and angular speed of GNP versus time. The dash line: with Brownian motion; the solid line: without Brownian motion.

**Reference**

1. Volpe, G. & Volpe, G. Simulation of a Brownian particle in an optical trap. *Am. J. Phys.* **81**(3), 224-30 (2013).
2. Baffou, G., Berto, P., Bermúdez Ureña, E., Quidant, R., Monneret, S., Polleux, J. & Rigneault, H. Photoinduced heating of nanoparticle arrays. *ACS Nano* **7**(8), 6478-88 (2013).
3. Liaw, J.-W., Huang, C.-W., Huang, M.-C. & Kuo, M.-K. Plasmon-enhanced optical bending and heating on V-shaped deformation of gold nanorod. *Appl. Phys. A* **124**, 17 (2018).
4. Liaw, J.-W., Liu, G., Ku, Y.-C. & Kuo, M.-K. Plasmon-enhanced photothermal and optomechanical deformations of a gold nanoparticle. *Nanomaterials* **10**, 1881 (2020).
